# Wearable accelerometer-derived physical activity and incident disease

**DOI:** 10.1038/s41746-022-00676-9

**Published:** 2022-09-02

**Authors:** Shaan Khurshid, Lu-Chen Weng, Victor Nauffal, James P. Pirruccello, Rachael A. Venn, Mostafa A. Al-Alusi, Emelia J. Benjamin, Patrick T. Ellinor, Steven A. Lubitz

**Affiliations:** 1 Cardiovascular Research Center, Massachusetts General Hospital, Boston MA, USA; 2 Cardiovascular Disease Initiative, Broad Institute of Harvard and the Massachusetts Institute of Technology, Cambridge MA, USA; 3 Demoulas Center for Cardiac Arrhythmias, Massachusetts General Hospital, Boston, MA USA; 4 Division of Cardiology, Brigham and Women’s Hospital, Boston, MA USA; 5 Division of Cardiology, Massachusetts General Hospital, Boston, MA USA; 6 Boston University School of Medicine and School of Public Health, Boston, MA USA; 7 Framingham Heart Study, Framingham, MA USA

**Keywords:** Risk factors, Prognostic markers

## Abstract

Physical activity is regarded as favorable to health but effects across the spectrum of human disease are poorly quantified. In contrast to self-reported measures, wearable accelerometers can provide more precise and reproducible activity quantification. Using wrist-worn accelerometry data from the UK Biobank prospective cohort study, we test associations between moderate-to-vigorous physical activity (MVPA) – both total MVPA minutes and whether MVPA is above a guideline-based threshold of ≥150 min/week—and incidence of 697 diseases using Cox proportional hazards models adjusted for age, sex, body mass index, smoking, Townsend Deprivation Index, educational attainment, diet quality, alcohol use, blood pressure, anti-hypertensive use. We correct for multiplicity at a false discovery rate of 1%. We perform analogous testing using self-reported MVPA. Among 96,244 adults wearing accelerometers for one week (age 62 ± 8 years), MVPA is associated with 373 (54%) tested diseases over a median 6.3 years of follow-up. Greater MVPA is overwhelmingly associated with lower disease risk (98% of associations) with hazard ratios (HRs) ranging 0.70–0.98 per 150 min increase in weekly MVPA, and associations spanning all 16 disease categories tested. Overall, associations with lower disease risk are enriched for cardiac (16%), digestive (14%), endocrine/metabolic (10%), and respiratory conditions (8%) (chi-square *p* < 0.01). Similar patterns are observed using the guideline-based threshold of ≥150 MVPA min/week. Some of the strongest associations with guideline-adherent activity include lower risks of incident heart failure (HR 0.65, 95% CI 0.55–0.77), type 2 diabetes (HR 0.64, 95% CI 0.58–0.71), cholelithiasis (HR 0.61, 95% CI 0.54–0.70), and chronic bronchitis (HR 0.42, 95% CI 0.33–0.54). When assessed within 456,374 individuals providing self-reported MVPA, effect sizes for guideline-adherent activity are substantially smaller (e.g., heart failure HR 0.84, 95% CI 0.80–0.88). Greater wearable device-based physical activity is robustly associated with lower disease incidence. Future studies are warranted to identify potential mechanisms linking physical activity and disease, and assess whether optimization of measured activity can reduce disease risk.

## Introduction

Physical activity may have important health benefits^1^, though the effects of physical activity across the range of human disease are poorly quantified. Examining relations between activity and disease risk may provide a comprehensive understanding of the benefits of physical activity, a modifiable lifestyle behavior. Past studies have generally assessed physical activity using self-report data^2–5^, which are subject to recall bias and correlate only modestly with measured energy expenditure^6^. Studies that have measured activity using wearable sensors have commonly quantified proprietary step counts^7^ or continuous acceleration^8,9^, which may be difficult to contextualize in terms of consensus activity recommendations, which typically recommend specific quantities of moderate-to-vigorous physical activity (MVPA)^1,10,11^. Past studies have also typically assessed only a limited set of outcomes (e.g., mortality)^9,12^, or focused only on prevalent disease^13^.

To address these challenges, we examine a unique, large prospective cohort study, the UK Biobank, which comprises over 90,000 individuals wearing wrist-worn triaxial accelerometers for one week. Our aim is to systematically identify associations between physical activity and a comprehensive array of incident human diseases, both to inform future research investigating potential causal mechanisms and to guide preventive efforts leveraging physical activity to reduce disease incidence. The use of wearable accelerometer-based physical activity measurements allows for precise and reproducible^8^ ascertainment of physical activity, quantified as minutes of MVPA and also classified into binary categories divided at a guideline-recommended threshold of ≥150 min of MVPA per week^1,10,11^. Utilizing linkage to national health records, we comprehensively assess associations between measured activity and longitudinal incidence of roughly 700 conditions spanning the full spectrum of human disease. We observe that device-measured activity is associated with lower risk of more than 350 incident conditions spanning the full spectrum of human disease, and that measured activity is a stronger indicator of disease risk as compared to self-reported activity obtained within the same population. Our findings will inform future work to identify mechanisms linking physical activity and disease, and suggest that efforts to optimize measured physical activity may result in lower disease incidence.

## Results

### Measured activity sample

**Fig. 1:**
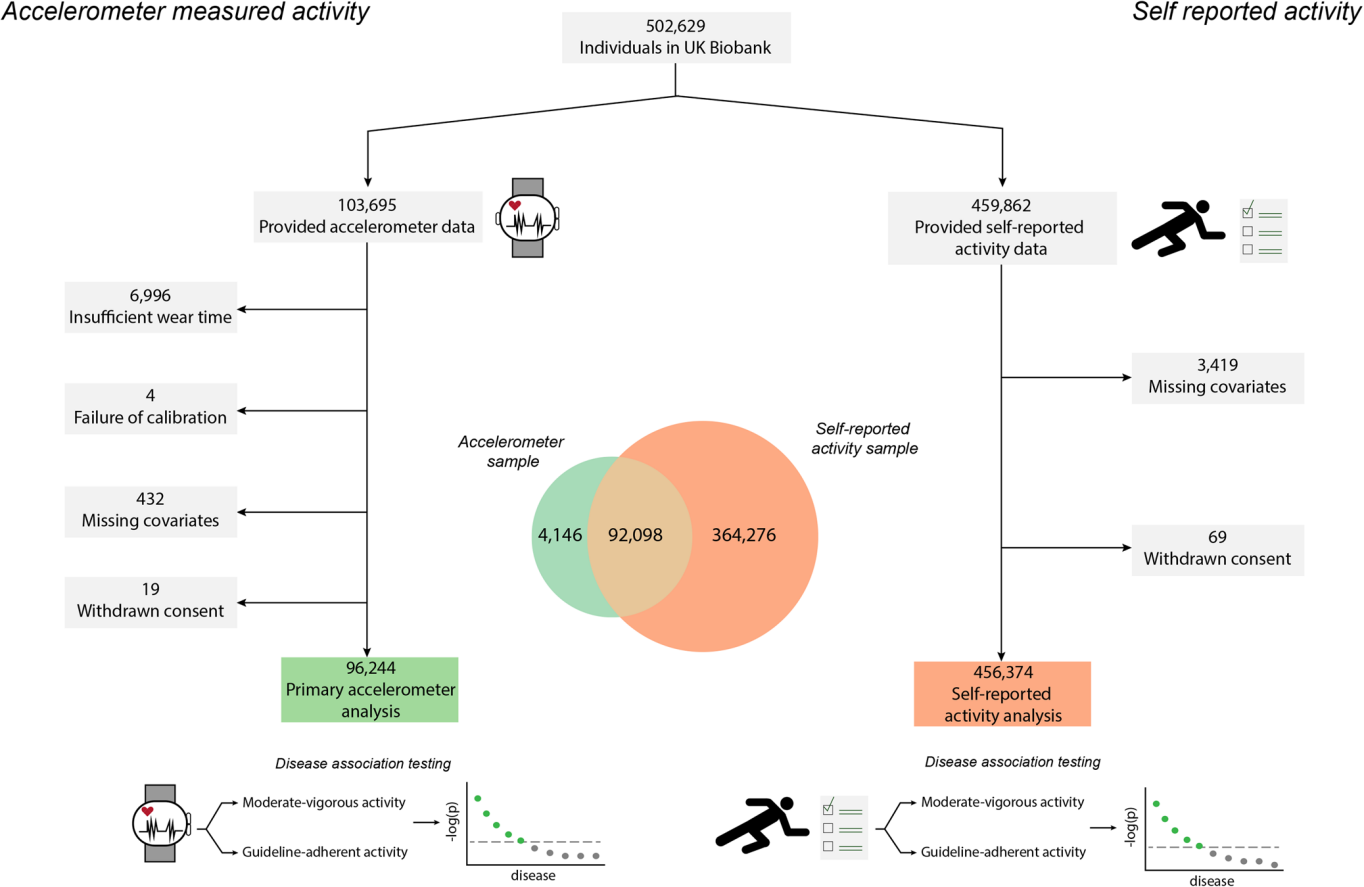
Study overview. Depicted is a graphical overview of the study. Within 96,244 UK Biobank participants who wore a wrist-worn accelerometer for one week, we tested for associations between objectively measured moderate-to-vigorous physical activity and 697 incident diseases. We performed analogous association testing in 456,374 UK Biobank participants who provided self-reported activity data. There was overlap between the two analysis samples, as depicted by the Venn diagram in the center.

After removing individuals whose accelerometer measurements failed quality control metrics, we performed disease association testing in 96,244 individuals (Fig. 1). The mean age was 62 ± 8 years and 56% were female. Individuals had a median MVPA of 135 min/week (quartile 1:60, quartile 3:250) and 46% of individuals achieved guideline-recommended levels. MVPA distributions are shown in Supplementary Fig. 1. Detailed sample characteristics are shown in Table 1.

**Table 1:** Baseline characteristics of study samples.

Baseline characteristic^a^	Wrist-worn accelerometer sample (*N* = 96,244)	Self-reported activity sample (*N* = 456,374)^b^
Mean ± SD*,* median *(quartile 1, quartile 3), or N (%)*		
Age	62.4 ± 7.8	56.9 ± 8.1
Female sex	54,169 (56.3%)	244,928 (53.7%)
Ethnic background^c^		
White	93,001 (96.6%)	433,281 (94.9%)
Asian	1114 (1.2%)	8829 (1.9%)
Black	805 (0.8%)	6512 (1.4%)
Mixed	526 (0.5%)	2646 (0.6%)
Other/Unknown	798 (0.8%)	5106 (1.1%)
Tobacco		
Current	6478 (6.7%)	46,337 (10.2%)
Former	34,655 (36.0%)	158,696 (34.8%)
Never	55,111 (57.3%)	251,341 (55.1%)
Alcohol intake (g/week)	96 (16, 176)	80 (8, 176)
Townsend Deprivation Index	−1.7 ± 2.8	−1.4 ± 3.0
Educational attainment (years)	15.3 ± 4.7	14.1 ± 4.9
Diet quality		
Poor	30,887 (32.1%)	156,690 (34.3%)
Intermediate	48,290 (50.2%)	226,816 (49.7%)
Good	17,067 (17.7%)	72,868 (16.0%)
Anti-hypertensive medication use	16,797 (17.5%)	91,143 (20.0%)
Body mass index (kg/m^2^)	26.7 ± 4.5	27.3 ± 4.7
Systolic blood pressure (mmHg)	137 ± 18	138 ± 19
Diastolic blood pressure (mmHg)	82 ± 10	82 ± 10
Hypertension	27,085 (28.1%)	130,357 (28.6%)
Diabetes	3,196 (3.3%)	11,552 (2.5%)

*SD* standard deviation.

^a^Baseline characteristics defined at end accelerometer wear for acceleration sample, and at enrollment for the self-reported activity sample.

^b^There is overlap between the accelerometer sample and the self-reported activity sample (see Fig. 1).

^c^Self-reported ethnic background.

### Associations between measured activity and incident disease

**Fig. 2:**
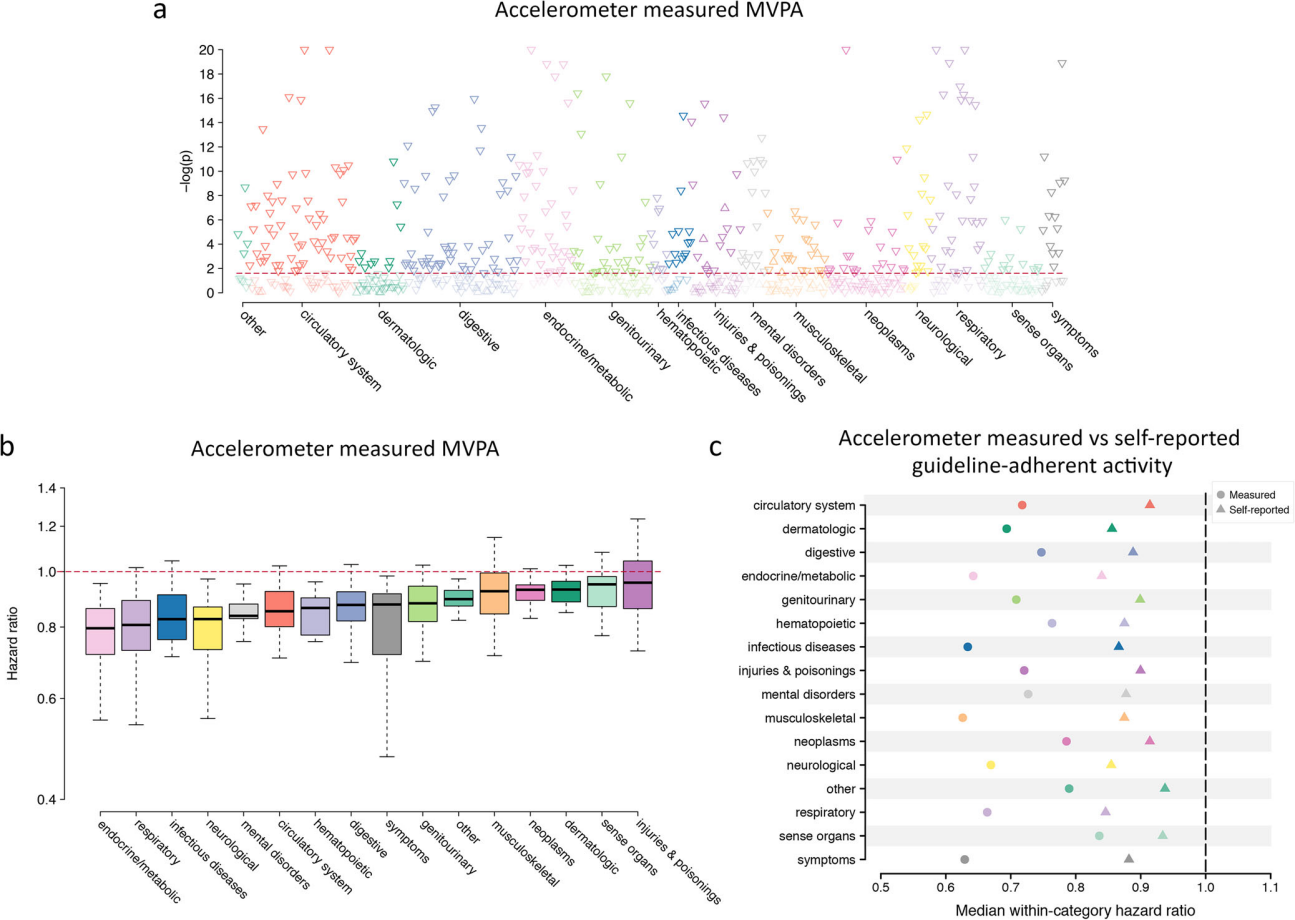
Accelerometer-measured physical activity and incident disease. **a** Plots the negative log10 *p* value for the association between accelerometer-measured moderate-to-vigorous physical activity (MVPA) and incident disease (grouped by category on the *x*-axis) in Cox proportional hazards models adjusted for age, sex, body mass index, Townsend Deprivation Index, smoking status, alcohol use, anti-hypertensive medication use, systolic blood pressure, and diastolic blood pressure, with darker shaded points meeting significance at a false discovery rate (FDR) of 1% (horizontal red line). Upward-facing triangles represent higher risk (hazard ratios >1), while downward-facing triangles represent lower risk (hazard ratio <1). *P* values smaller than 1 × 10^−20^ are displayed as 1 × 10^−20^ for graphical purposes. **b** Shows the distribution of hazard ratios per 150-min increase in MVPA per week observed across each disease category (*x*-axis), with the center line depicting the within-category median hazard ratio, the bounds of the box representing quartile 1 to quartile 3, and the whiskers extending 1.5 interquartile ranges beyond the box. Categories are arranged by increasing median hazard ratio, from lowest (left) to highest (right). **c** Compares the median within-category hazard ratio observed with guideline-adherent activity (i.e., ≥150 min of MVPA per week^1,10,11^) defined using accelerometer-measured (circles) versus self-reported (triangles) MVPA. Included in the comparison are 323 diseases for which there was a nominally significant association (*p* < 0.05) with both exposure definitions and an effect size suggesting a lower risk of disease.

At a median follow-up of 6.2 years (quartile 1:5.7, quartile 3:6.7), MVPA was associated with risk of 373 out of 697 (54%) incident diseases tested at an FDR of 1% (Fig. 2a). Of the significant associations, 367 (98%) indicated a lower risk of disease with greater MVPA (hazard ratio [HR] range 0.70–0.98 per 150-min increase in weekly MVPA). Some of the strongest associations included lower risks of atherosclerosis (HR 0.57, 95% CI 0.44–0.74), type 2 diabetes (HR 0.74, 95% CI 0.70–0.79), chronic bronchitis (HR 0.44, 95% CI 0.37–0.53), and depression (HR 0.84, 95% CI 0.79–0.88) (Supplementary Fig. 2). Among all significant associations with lower disease risk, most conditions represented cardiac (16%), digestive (14%), endocrine/metabolic (10%), and respiratory diseases (8%), although associations were observed in all categories tested (chi-square *p* < 0.01, Supplementary Figs. 3 and 4). The distribution of effect sizes varied by disease category, with the lowest median hazard ratios (i.e., largest effects) observed for endocrine/metabolic, respiratory, and infectious diseases (Fig. 2b).

There were six associations between greater MVPA and higher risk of disease (HR range 1.08–1.24), and all represented injuries/poisonings, musculoskeletal, and dermatologic conditions. Examples included greater risk of disorders of muscle, ligament, and fascia (HR 1.09, 95% CI 1.03–1.15) and fracture of the radius or ulna (HR 1.09, 95% CI 1.02–1.15).

**Fig. 3:**
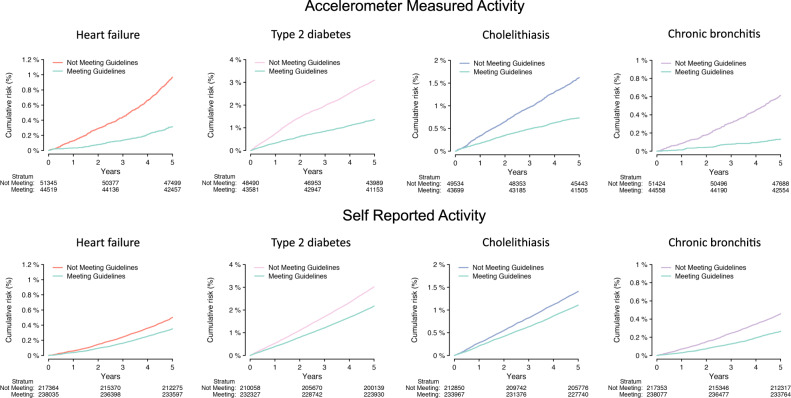
Cumulative risk of disease stratified by guideline-adherent physical activity. Depicted is the 5-year cumulative risk of heart failure, type 2 diabetes, cholelithasis, and chronic bronchitis, stratified guideline-adherent activity according to accelerometer-measured moderate-to-vigorous physical activity (MVPA, top panels) and self-reported MVPA (bottom panels). In each plot, individuals are grouped into binary categories according to the guideline-based threshold of ≥150 min of MVPA/week. Red, pink, blue, and purple strata represent individuals meeting guideline-based levels, and the teal stratum represents individuals not meeting guideline-based levels^1,10,11^. In each plot, the number remaining at risk over time is depicted below. Representative diseases were selected from the four categories having the greatest enrichment for associations with activity, where each disease was significantly associated with both accelerometer-measured and self-reported activity at a false discovery rate of 1%.

Overall, we obtained similar results when categorizing MVPA at the ≥150 min per week threshold recommended in consensus guidelines^1,10,11^ (306 associations with lower risk of disease, HR range 0.11–0.91, Supplementary Fig. 5). Some of the strongest associations with guideline-based physical activity included lower risks of heart failure (HR 0.65, 95% CI 0.55–0.77), type 2 diabetes (HR 0.64, 95% CI 0.58–0.71), cholelithiasis (HR 0.61, 95% CI 0.54–0.70), and chronic bronchitis (HR 0.42, 95% CI 0.33–0.54). Plots of the 5-year cumulative risk of these four conditions demonstrated consistent and substantial separation of longitudinal disease incidence on the basis of accelerometer-derived guideline-adherent activity (Fig. 3). Multivariable adjusted cumulative risk curves for men and women were similar and are shown in Supplementary Figs. 6 and 7.

**Fig. 4:**
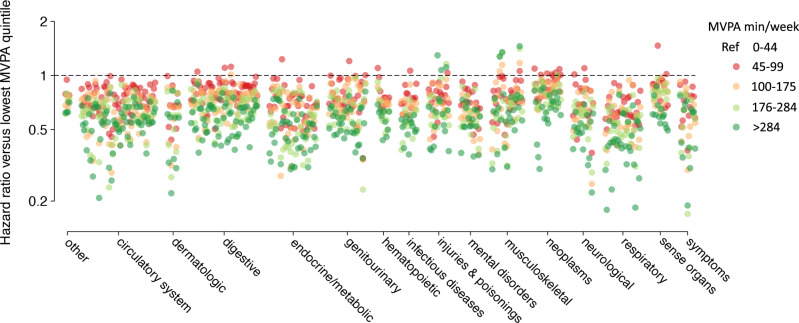
Associations between quintile of measured MVPA and incident disease. Depicted is the relative hazard of incident disease according to quintile of accelerometer-measured moderate-to-vigorous physical activity (MVPA) and grouped by disease category. Each disease is represented by four points, with each point representing the hazard ratio associated with a given quintile of MVPA (quintile 2 = red, quintile 3 = orange, quintile 4 = light green, quintile 5 = dark green), as compared to the lowest quintile (quintile 1) as the referent. MVPA volumes corresponding to each quintile are shown in the legend. The hashed horizontal line depicts a hazard ratio of one (i.e., equal hazard to quintile 1). A single value below 0.1 was rounded to 0.1 for graphical purposes (bottom right).

For most conditions, risk of disease was lowest at higher MVPA levels (Fig. 4 and Supplementary Fig. 8). Nevertheless, for a few conditions enriched within certain disease categories (e.g., musculoskeletal, injuries/poisonings), risk was lowest at intermediate MVPA levels. The pattern of associations observed for alternative MVPA thresholds (i.e., ≥75 and ≥300 MVPA min/week) was generally similar to that seen at the ≥150 min per week threshold, although the total number of significant associations at ≥300 MVPA min/week was smaller (Supplementary Fig. 9).

### Self-reported activity

Within 456,374 UK Biobank participants providing questionnaire-based activity data (Table 1 and Fig. 1), self-reported MVPA was also generally associated with lower risk of disease, although there were fewer associations overall (Supplementary Figs. 3, 4, 10, and 11). As compared to accelerometer-derived MVPA, self-reported MVPA had several more associations indicating a higher risk of disease, enriched for musculoskeletal conditions (e.g., localized osteoarthrosis, HR 1.02, 95% CI 1.01–1.04) and injuries/poisonings (e.g., joint/ligament sprain, HR 1.02, 95% CI 1.02–1.03) (Supplementary Figs. 3,4, 10, and 11). When assessing guideline-adherent activity, many of the associations observed with accelerometer-derived activity were again seen, but effect sizes were smaller. For example, self-reported guideline-adherent activity was also associated with lower risks of heart failure (HR 0.84, 95% CI 0.80–0.88), type 2 diabetes (HR 0.85, 95% CI 0.83–0.87), cholelithiasis (HR 0.86, 95% CI 0.84–0.89), and chronic bronchitis (HR 0.70, 95% CI 0.67–0.75) (Figs. 2c and 3). Association results for diseases related to both accelerometer-measured and self-reported guideline-adherent activity are summarized in Table 2.

**Table 2:** Diseases associated with at least 150 min per week of moderate-to-vigorous physical activity.

	*Measured MVPA*					*Self-reported MVPA*				
Disease^a^	*N* events	Hazard ratio (95% CI)	*p*	*E*_point_^b^	*E*_null_^c^	*N* events	Hazard ratio (95% CI)	*p*	*E*_point_^b^	*E*_null_^C^
Circulatory system										
Hypertension	9603	0.81 (0.78–0.85)	4.78 × 10^–21^	1.77	1.64	94357	0.94 (0.93–0.95)	1.62 × 10^–21^	1.26	1.23
Other disorders of circulatory system	3072	0.73 (0.67–0.79)	5.11 × 10^–15^	2.09	1.85	27412	0.95 (0.92–0.97)	4.21 × 10^–06^	1.31	1.22
Cerebrovascular disease	1665	0.70 (0.62–0.78)	7.32 × 10^–11^	2.23	1.90	15232	0.92 (0.89–0.95)	4.15 × 10^–07^	1.39	1.29
Hypotension	1770	0.71 (0.64–0.79)	1.24 × 10^–10^	2.17	1.86	15008	0.86 (0.84–0.89)	5.19 × 10^–19^	1.59	1.49
Peripheral vascular disease, unspecified	340	0.42 (0.32–0.56)	9.78 × 10^–10^	4.17	2.99	4146	0.81 (0.76–0.86)	1.22 × 10^–11^	1.78	1.60
Dermatologic										
Chronic ulcer of skin	484	0.58 (0.47–0.72)	8.04 × 10^–07^	2.85	2.13	5350	0.79 (0.75–0.83)	1.42 × 10^–17^	1.85	1.69
Chronic ulcer of leg or foot	180	0.43 (0.29–0.64)	2.57 × 10^–05^	4.07	2.51	2162	0.75 (0.69–0.82)	1.51 × 10^–10^	1.99	1.74
Cellulitis and abscess of arm/hand	729	0.75 (0.63–0.88)	6.42 × 10^–04^	2.01	1.52	7571	0.92 (0.88–0.97)	7.94 × 10^–04^	1.38	1.22
Decubitus ulcer	308	0.64 (0.49–0.84)	1.03 × 10^–03^	2.50	1.68	3428	0.78 (0.73–0.83)	7.68 × 10^–13^	1.89	1.69
Diffuse diseases of connective tissue	147	0.56 (0.38–0.81)	2.22 × 10^–03^	2.99	1.77	1388	0.77 (0.69–0.85)	1.29 × 10^–06^	1.93	1.62
Digestive										
Diseases of esophagus	5194	0.79 (0.74–0.84)	6.61 × 10^–15^	1.85	1.68	48077	0.94 (0.92–0.96)	6.05 × 10^–11^	1.32	1.26
Diverticulosis	5546	0.80 (0.75–0.85)	2.32 × 10^–14^	1.81	1.65	44157	0.95 (0.94–0.97)	6.62 × 10^–07^	1.28	1.20
Cholelithiasis	1352	0.61 (0.54–0.70)	2.33 × 10^–14^	2.64	2.23	13966	0.86 (0.84–0.89)	4.15 × 10^–17^	1.58	1.48
Esophagitis, GERD and related diseases	4920	0.79 (0.74–0.84)	8.75 × 10^–14^	1.84	1.66	45075	0.94 (0.93–0.96)	6.22 × 10^–10^	1.32	1.25
Cholelithiasis and cholecystitis	1585	0.65 (0.58–0.73)	2.62 × 10^–13^	2.43	2.07	16048	0.88 (0.85–0.91)	1.36 × 10^–15^	1.53	1.44
Endocrine/metabolic system										
Type 2 diabetes	2043	0.64 (0.58–0.71)	4.04 × 10^–17^	2.50	2.17	24501	0.85 (0.83–0.87)	2.91 × 10^–34^	1.62	1.55
Diabetes mellitus	2509	0.68 (0.62–0.75)	4.89 × 10^–16^	2.29	2.01	28670	0.86 (0.84–0.88)	1.11 × 10^–33^	1.58	1.51
Disorders of fluid, electrolyte, and acid-base balance	2363	0.71 (0.65–0.78)	3.59 × 10^–13^	2.17	1.89	21222	0.84 (0.82–0.86)	7.26 × 10^–36^	1.67	1.59
Hyperlipidemia	5021	0.83 (0.78–0.89)	4.83 × 10^–09^	1.69	1.51	50994	0.94 (0.92–0.95)	2.63 × 10^–13^	1.34	1.28
Vitamin deficiency	1023	0.66 (0.57–0.76)	5.02 × 10^–09^	2.41	1.97	10602	0.84 (0.81–0.87)	1.49 × 10^–18^	1.66	1.55
Genitourinary system										
Renal failure	3294	0.74 (0.68–0.80)	7.03 × 10^–14^	2.05	1.81	27750	0.88 (0.86–0.90)	1.29 × 10^–24^	1.52	1.45
Urinary tract infection	2030	0.69 (0.63–0.77)	5.29 × 10^–13^	2.24	1.93	26051	0.91 (0.89–0.93)	6.99 × 10^–14^	1.43	1.35
Acute renal failure	1870	0.68 (0.61–0.76)	2.65 × 10^–12^	2.29	1.96	16511	0.85 (0.83–0.88)	2.72 × 10^–24^	1.63	1.54
Chronic renal failure	1809	0.68 (0.61–0.76)	7.54 × 10^–12^	2.32	1.97	15438	0.87 (0.84–0.90)	1.60 × 10^–17^	1.56	1.47
Chronic kidney disease, stage III	1195	0.64 (0.55–0.73)	3.71 × 10^–10^	2.51	2.07	9350	0.88 (0.85–0.92)	2.12 × 10^–09^	1.52	1.40
Hematopoietic system										
Other anemias	2053	0.75 (0.68–0.82)	3.79 × 10^–09^	2.01	1.73	19612	0.86 (0.84–0.89)	4.51 × 10^–25^	1.60	1.51
Iron deficiency anemias	1757	0.78 (0.70–0.87)	3.43 × 10^–06^	1.88	1.57	15308	0.88 (0.85–0.90)	8.87 × 10^–16^	1.54	1.45
Purpura and other hemorrhagic conditions	433	0.67 (0.54–0.83)	2.63 × 10^–04^	2.35	1.70	4342	0.88 (0.82–0.93)	1.65 × 10^–05^	1.54	1.36
Thrombocytopenia	362	0.66 (0.52–0.83)	4.52 × 10^–04^	2.41	1.70	3185	0.86 (0.81–0.93)	4.90 × 10^–05^	1.58	1.37
Diseases of white blood cells	729	0.81 (0.69–0.95)	1.09 × 10^–02^	1.76	1.27	6394	0.92 (0.88–0.97)	1.24 × 10^–03^	1.39	1.22
Infectious diseases										
Septicemia	1177	0.61 (0.54–0.70)	5.82 × 10^–13^	2.65	2.21	10809	0.84 (0.81–0.87)	7.71 × 10^–19^	1.66	1.55
Bacterial infection, NOS	1473	0.69 (0.61–0.77)	4.00 × 10^–10^	2.26	1.91	15526	0.87 (0.84–0.89)	9.97 × 10^–19^	1.58	1.48
* E. coli*	572	0.61 (0.50–0.74)	4.52 × 10^–07^	2.68	2.05	5569	0.82 (0.78–0.87)	5.30 × 10^–13^	1.73	1.58
Intestinal infection	446	0.64 (0.52–0.79)	3.22 × 10^–05^	2.51	1.85	6319	0.93 (0.89–0.98)	5.81 × 10^–03^	1.35	1.17
Gram negative septicemia	282	0.57 (0.43–0.75)	5.22 × 10^–05^	2.93	2.02	2305	0.87 (0.80–0.94)	7.49 × 10^–04^	1.57	1.32
Injuries and poisonings										
Sepsis and SIRS	1130	0.61 (0.53–0.70)	6.72 × 10^–13^	2.68	2.23	10286	0.84 (0.80–0.87)	5.57 × 10^–19^	1.68	1.56
Personal history of allergy to medicinal agents	1858	0.68 (0.61–0.76)	7.77 × 10^–13^	2.29	1.97	14174	0.94 (0.91–0.97)	2.31 × 10^–04^	1.33	1.21
Sepsis	1115	0.61 (0.53–0.70)	1.66 × 10^–12^	2.66	2.21	10177	0.84 (0.80–0.87)	1.27 × 10^–18^	1.68	1.56
Effects radiation, NOS	1348	0.76 (0.67–0.85)	3.93 × 10^–06^	1.97	1.62	9383	0.92 (0.88–0.96)	4.69 × 10^–05^	1.40	1.26
Complications of transplants and reattached limbs	2255	0.82 (0.74–0.89)	1.16 × 10^–05^	1.75	1.48	21690	0.96 (0.93–0.98)	2.09 × 10^–03^	1.26	1.14
Mental disorders										
Mood disorders	1957	0.71 (0.64–0.78)	6.94 × 10^–12^	2.17	1.88	22842	0.88 (0.86–0.90)	5.83 × 10^–22^	1.54	1.46
Depression	1935	0.71 (0.64–0.78)	7.70 × 10^–12^	2.18	1.88	22482	0.88 (0.85–0.90)	2.27 × 10^–22^	1.54	1.46
Anxiety disorders	2061	0.72 (0.65–0.79)	1.24 × 10^–11^	2.13	1.84	18341	0.91 (0.88–0.93)	3.14 × 10^–11^	1.44	1.35
Other mental disorder	6792	0.85 (0.81–0.90)	1.00 × 10^–09^	1.63	1.48	59516	0.95 (0.94–0.97)	1.35 × 10^–08^	1.27	1.21
Tobacco use disorder	1191	0.72 (0.64–0.82)	4.36 × 10^–07^	2.11	1.74	19461	0.94 (0.92–0.97)	4.00 × 10^–05^	1.32	1.21
Musculoskeletal										
Osteoporosis, NOS	1433	0.75 (0.67–0.84)	7.43 × 10^–07^	2.00	1.67	12265	0.84 (0.81–0.87)	4.65 × 10^–21^	1.66	1.55
Other disorders of bone and cartilage	971	0.72 (0.62–0.82)	2.93 × 10^–06^	2.13	1.72	8029	0.93 (0.89–0.98)	2.13 × 10^–03^	1.35	1.19
Rheumatoid arthritis	498	0.63 (0.52–0.78)	1.34 × 10^–05^	2.54	1.89	4684	0.88 (0.83–0.93)	2.26 × 10^–05^	1.52	1.34
Curvature of spine	238	0.51 (0.37–0.69)	1.90 × 10^–05^	3.33	2.24	1802	0.84 (0.76–0.92)	1.64 × 10^–04^	1.68	1.40
Peripheral enthesopathies and allied syndromes	1854	0.81 (0.73–0.89)	2.17 × 10^–05^	1.79	1.49	22736	1.05 (1.02–1.08)	2.91 × 10^–04^	1.28	1.17
Neoplasms										
Acquired absence of breast	4230	0.77 (0.72–0.82)	1.30 × 10^–14^	1.93	1.73	30544	0.93 (0.91–0.95)	4.63 × 10^–10^	1.36	1.28
Chemotherapy	4145	0.77 (0.72–0.83)	7.00 × 10^–14^	1.91	1.71	48201	0.93 (0.92–0.95)	3.45 × 10^–13^	1.34	1.28
Benign neoplasm of other parts of digestive system	1225	0.69 (0.61–0.78)	7.82 × 10^–09^	2.27	1.88	9432	0.85 (0.82–0.89)	6.05 × 10^–14^	1.62	1.49
Benign neoplasm of colon	4044	0.86 (0.81–0.92)	1.70 × 10^–05^	1.58	1.38	32432	0.94 (0.92–0.96)	2.43 × 10^–08^	1.33	1.25
Cancer within the respiratory system	499	0.69 (0.56–0.84)	3.50 × 10^–04^	2.27	1.65	5482	0.87 (0.82–0.91)	1.31 × 10^–07^	1.58	1.42
Neurological										
Other CNS infection and poliomyelitis	2084	0.70 (0.64–0.77)	6.82 × 10^–13^	2.21	1.91	15745	0.92 (0.89–0.95)	7.55 × 10^–08^	1.41	1.30
Abnormal movement	1138	0.63 (0.55–0.73)	5.11 × 10^–11^	2.54	2.10	12420	0.85 (0.82–0.89)	7.08 × 10^–18^	1.62	1.51
Abnormality of gait	818	0.58 (0.49–0.68)	1.37 × 10^–10^	2.85	2.28	7241	0.78 (0.74–0.81)	4.24 × 10^–26^	1.90	1.76
Parkinson’s disease	284	0.50 (0.38–0.66)	9.90 × 10^–07^	3.39	2.39	2709	0.84 (0.78–0.91)	6.37 × 10^–06^	1.67	1.44
Sleep disorders	850	0.69 (0.59–0.81)	3.38 × 10^–06^	2.26	1.78	10045	0.88 (0.84–0.91)	2.37 × 10^–10^	1.53	1.41
Other										
Other tests	6647	0.86 (0.81–0.90)	2.87 × 10^–09^	1.61	1.46	55345	0.96 (0.94–0.97)	6.11 × 10^–07^	1.26	1.19
Symptoms concerning nutrition, metabolism, and development	1231	0.76 (0.68–0.86)	1.60 × 10^–05^	1.94	1.58	10412	0.86 (0.83–0.89)	2.32 × 10^–14^	1.60	1.48
Respiratory system										
Chronic airway obstruction	1730	0.58 (0.52–0.65)	9.29 × 10^–22^	2.86	2.47	18354	0.84 (0.81–0.86)	1.51 × 10^–32^	1.68	1.59
Pneumonia	2366	0.69 (0.63–0.76)	2.33 × 10^–15^	2.26	1.98	22083	0.85 (0.83–0.87)	1.67 × 10^–33^	1.64	1.56
*Pneumococcal pneumonia*	1443	0.64 (0.57–0.72)	1.88 × 10^–13^	2.50	2.12	12790	0.84 (0.81–0.87)	1.15 × 10^–22^	1.67	1.57
Other diseases of respiratory system, NEC	1551	0.65 (0.58–0.73)	1.92 × 10^–13^	2.44	2.08	14497	0.89 (0.87–0.92)	3.27 × 10^–11^	1.48	1.38
Bacterial pneumonia	1514	0.65 (0.58–0.73)	5.45 × 10^–13^	2.43	2.07	13496	0.84 (0.82–0.87)	2.93 × 10^–22^	1.65	1.55
Sense organs										
Dizziness and giddiness	882	0.78 (0.68–0.90)	9.18 × 10^–04^	1.88	1.45	7687	0.93 (0.89–0.97)	1.11 × 10^–03^	1.37	1.21
Vertiginous syndromes and other vestibular disorders	1323	0.82 (0.73–0.93)	1.32 × 10^–03^	1.72	1.37	12432	0.94 (0.91–0.98)	1.02 × 10^–03^	1.32	1.18
Cataract	5676	0.93 (0.88–0.99)	1.72 × 10^–02^	1.35	1.12	42114	0.93 (0.92–0.95)	8.80 × 10^–12^	1.34	1.28
Symptoms										
Symptoms involving nervous and musculoskeletal systems	1268	0.60 (0.53–0.69)	5.15 × 10^–14^	2.70	2.26	11216	0.82 (0.79–0.85)	1.71 × 10^–25^	1.74	1.63
Malaise and fatigue	752	0.58 (0.49–0.69)	1.42 × 10^–10^	2.82	2.27	6435	0.83 (0.79–0.87)	4.69 × 10^–13^	1.69	1.55
Myalgia and myositis, unspecified	435	0.50 (0.40–0.62)	1.34 × 10^–09^	3.45	2.59	4212	0.86 (0.80–0.91)	6.29 × 10^–07^	1.61	1.43
Edema	555	0.59 (0.48–0.72)	3.30 × 10^–07^	2.78	2.11	6254	0.85 (0.81–0.90)	1.22 × 10^–09^	1.62	1.47
Nausea and vomiting	1703	0.76 (0.69–0.85)	4.94 × 10^–07^	1.95	1.64	15735	0.94 (0.91–0.97)	2.22 × 10^–04^	1.32	1.20

*GERD* gastro-esophageal reflux disease, *NOS* not otherwise specified, *SIRS* systemic inflammatory response syndrome, *CNS* central nervous system, *NEC* not elsewhere classified.

^a^Displayed are the top five (when present) diseases with each category having the strongest statistical associations with guideline-adherent measured MVPA, among diseases associated with both measured and self-reported guideline-adherent MVPA at a false discovery rate of 1% (see text). For display purposes, disease entities represented multiple times (e.g., hypertension, essential hypertension) are shown only once (unabridged results in Supplementary Data).

^b^*E*-value for the point estimate. The *E*-value is defined as the minimum strength of association on the risk ratio scale that an unadjusted confounder would need to have with the exposure and the outcome to nullify the observed association. Higher values represent a lower likelihood the observed association is due to confounding.

^c^ *E*-value for the bound closest to the null.

### Secondary analyses

We repeated disease association testing using overall mean acceleration, which demonstrated a similar pattern of associations as accelerometer-derived MVPA (Supplementary Fig. 12). Greater vigorous activity was also generally associated with lower disease risk, although the total number of associations was smaller (Supplementary Figs. 13–15). The observed pattern of associations between measured MVPA and incident disease was consistent across subgroups of age (i.e., age <55, age 55–64, and age ≥65, Supplementary Fig. 16). Associations with measured MVPA were similar when only hospital data (i.e., excluding general practitioner data) were used to define outcomes (*n* = 343 significant associations, Supplementary Fig. 17). There were a greater number of significant associations in models without BMI, blood pressure, or anti-hypertensive use as covariates (*n* = 500, Supplementary Fig. 18). In a landmark analysis in which person-time began two years after accelerometer wear, associations with measured MVPA were similar, although the total number was somewhat smaller (*n* = 259 significant associations, Supplementary Fig. 19). Disease-level association results from the primary and secondary analyses are available in the Supplementary Data.

## Discussion

In summary, within over 90,000 individuals wearing a wrist-based activity sensor over the course of one week, we quantified associations between measured physical activity (quantified as MVPA) and future risk of nearly 700 diseases. We observed strong associations between physical activity and hundreds of conditions enriched for cardiac, digestive, endocrine/metabolic, respiratory and other diseases. Achievement of guideline-adherent activity levels was overwhelmingly associated with lower disease risk. At the same time, greater MVPA was associated with progressively lower disease risk both below and above guideline-based thresholds. Although self-reported MVPA was also associated with lower disease risk, both the total number of associations and magnitude of effect sizes were greater using accelerometer-measured MVPA. Overall, our results prioritize a number of diseases for future study to better define mechanisms by which physical activity may affect disease risk, and suggest that wearable sensors may be important tools for evaluating efforts to modify disease risk using physical activity.

Our findings support and extend previous observations linking physical activity with lower risk of disease. Multiple studies have demonstrated associations between greater physical activity and lower risk of disease, primarily cardiometabolic conditions^2,14^. Importantly, however, most previous studies have relied on self-reported data, which are subject to recall bias and measurement imprecision. In contrast, wearable accelerometers provide a mechanism for precise and reproducible activity measurement. Recently, accelerometer-measured physical activity has been associated with lower risks of cardiovascular^12^ and neurological disease^15^, as well as overall mortality^9^. Our findings extend prior results by quantifying associations with accelerometer-measured activity across a broad spectrum of disease, exploring physical activity dose-response relationships, and benchmarking the relative utility of device-measured versus self-reported activity as a marker of disease risk.

Our results suggest that achievement of guideline-recommended physical activity levels may be an important marker of substantially lower risk of a wide range of future diseases. Whether assessed as a continuous variable or dichotomized at a guideline-recommended threshold, greater MVPA was associated with lower risk of over 350 diseases. Although associations were enriched for cardiac, digestive, endocrine/metabolic, and respiratory conditions, the breadth of associations observed spanned every category tested. For example, individuals meeting guideline-recommended levels of accelerometer-derived MVPA had a 32% lower risk of an incident sleep disorder, which is consistent with randomized trial evidence supporting a beneficial role of exercise on sleep^16^. Similarly, guideline-recommended MVPA was associated with a 40% lower risk of cholelithiasis, which may be related to beneficial effects on gut motility^17^. Despite evaluating nearly 700 conditions, we observed only six significant associations in which activity was related to higher disease risk, with each representing a musculoskeletal disorder, injury, or dermatologic condition (e.g., corns and calluses), and potentially attributable to use-related degeneration.

Importantly, greater levels of measured activity—even if not to the point of achieving guideline-recommended levels—may be beneficial. When assessing dose-response relationships within the roughly 350 diseases significantly associated with measured MVPA, achievement of progressively higher quintiles of MVPA appeared monotonically associated with lower disease risk, with the lowest risk observed in the highest quintile. Similarly, we observed that achievement of activity at thresholds below (≥75 min of MVPA/week) and above (≥300 min of MVPA/week) the guideline-based threshold of ≥150 min^1,10,11^ were associated with lower disease risk. Importantly, current guidelines are largely based on self-reported activity data^1,10,11^, and the information content of accelerometer-derived activity may differ substantively from self-reported measures, potentially in a manner dependent upon which specific activities are being quantified^18^. As a result, future work is needed to define appropriate thresholds for accelerometer-derived activity. Nevertheless, our findings suggest that, in general, greater device-measured activity levels appear broadly associated with lower risks of most human diseases.

Our results suggest that wearable accelerometer-based activity measurement may play an important role in future public health efforts focused on physical activity. Prior reports have suggested that self-reported activity may be biased^5^, and therefore a weak surrogate for measured activity^6^. Indeed, recent evidence suggests that device-based activity measures may be a more powerful predictor of cardiovascular outcomes^19^ and mortality^5^ than self-reported activity within the same population. We similarly observed substantially stronger effect sizes and more substantial risk stratification when assessing guideline-adherent activity using accelerometer-derived as opposed to self-reported MVPA. Interestingly, although the distribution of significant associations was qualitatively similar using accelerometer-derived versus self-reported data, self-reported data suggested a considerably greater number of associations indicating greater disease risk, primarily comprising musculoskeletal conditions and injuries/poisonings. Future work is warranted to better understand potential differences in information content between measured and self-reported physical activity, and identify optimal methods for leveraging activity measurement to prevent disease.

Our study should be considered in the context of design. First, physical activity was measured for only one week. It is possible that a longer duration of monitoring would have led to more accurate classification of activity. Second, our comparisons between measured and self-reported activity may be susceptible to biases introduced by varying follow-up time and temporal differences in exposure ascertainment. Third, since our aim was to identify diseases for which physical activity may plausibly affect risk to generate hypotheses and inform future studies, we controlled for substantive confounders and took steps to explore reverse causality (e.g., 2-year blanking analysis). Nevertheless, we acknowledge that our findings reflect associations, that residual confounding and reverse causality may exist, and that robust causal inference analyses are warranted. Fourth, reliance on diagnosis codes to define outcomes introduces potential bias whereby individuals with lower physical activity levels may have more frequent healthcare contact leading to a higher likelihood of incident disease diagnosis. Fifth, although we performed data processing in accordance with previous practices using wrist-based accelerometers^8,20–22^, exposure classification in accelerometer-based studies may differ substantially based on processing choices made^23^. Sixth, in addition to overall MVPA, we assessed the guideline-recommended MVPA threshold of ≥150 min/week^1,10,11^ given that it is commonly used in clinical practice and has been associated with outcomes previously in accelerometer studies across multiple populations^21,22,24,25^. Nevertheless, we acknowledge that current activity guidelines are largely based on self-reported data, evidence to support a particular threshold of accelerometer-measured activity is limited, and optimal levels of device-measured activity may differ. Indeed, our findings generally suggest that greater MVPA levels are increasingly beneficial, whether below or above the 150 MVPA min/week threshold. Seventh, some of our subgroup analyses (e.g., age <55 years) may be underpowered due to lower event rates. Eighth, we report findings from a single community-based sample comprising comparatively healthy individuals from the UK^26^ whose activity habits may have been influenced by the awareness of being monitored, and therefore our results may not generalize to other populations.

In summary, we performed phenome-wide association testing for incident disease using a unique resource of accelerometer-measured physical activity obtained within over 90,000 individuals. We observed that device-measured activity—defined as continuous MVPA as well as according to guideline-based thresholds—is associated with lower risk of more than 350 incident conditions spanning the full spectrum of human disease. Measured activity was a stronger indicator of disease risk as compared to self-reported activity obtained within the same population. Our findings prioritize future work to identify potential mechanisms linking physical activity and disease, and suggest that optimization of measured physical activity levels may provide a mechanism to reduce future disease incidence.

## Methods

### Study population

The UK Biobank is a prospective cohort of 502,629 participants enrolled between 2006–2010^27^. Briefly, 9.2 million individuals aged 40–69 years living within 25 miles of 22 assessment centers in the UK were invited, and 5.4% participated in the baseline assessment. Questionnaires and physical measures were collected at recruitment, and all participants are followed for outcomes through linkage to national health-related datasets. All participants provided written informed consent. The UK Biobank was approved by the UK Biobank Research Ethics Committee (reference# 11/NW/0382). The use of UK Biobank data (application 17488) was approved by the local Mass General Brigham Health Institutional Review Board.

### Accelerometer-derived physical activity

Between February 2013–December 2015, 236,519 UK Biobank participants were invited to wear a wrist-worn accelerometer for one week, of whom 106,053 agreed to participate and 103,695 submitted data^8^. Participants were sent an Axivity AX3 (Newcastle upon Tyne, UK) wrist-worn triaxial accelerometer. The sensor captured continuous acceleration at 100 Hz with dynamic range of ±8 *g*.

As described previously, acceleration signals were calibrated to local gravity^8^. Sample data were combined into 5-s epochs, with each epoch represented by the average vector magnitude. Non-wear-time was identified as consecutive stationary episodes ≥60 min in duration in which all three axes had standard deviation <13.0 m*g*^28^. Epochs representing non-wear-time were imputed based on the average of similar time-of-day vector magnitude and intensity distribution data points on different days. We excluded individuals with insufficient wear-time to support imputation (<72 h of wear-time or no wear data in each one-hour period of the 24-h cycle), and whose signals were insufficient for calibration^8^.

The primary accelerometer-derived exposure was min of moderate-to-vigorous physical activity (MVPA), defined as the sum of 5-s epochs where mean acceleration was ≥100 m*g*^21,29^. As performed previously, we extracted MVPA in bouts (5-min periods where ≥80% of epochs met the MVPA threshold), which reduces misclassification of random wrist movement as MVPA^20,21^, and results in activity estimates in the UK Biobank which align more closely with expectations from UK population-based surveys^22^. We then classified whether MVPA levels met thresholds recommended in guidelines from the World Health Organization^10^, American College of Cardiology/American Heart Association^1^, and European Society of Cardiology^11^. Secondary exposures included overall mean acceleration, a surrogate for global physical activity that has been validated against energy expenditure^30^. For a minority of individuals (*n* = 2316, 2%) contributing >72 h but less than a full week of wear-time, we extrapolated the observed MVPA rate to one week to account for variable wear-time and to facilitate categorization of weekly guideline-based activity.

### Self-reported physical activity

Self-reported physical activity data were obtained at enrollment in over 450,000 UK Biobank participants using the short-form international physical activity questionnaire (IPAQ)^31^. We quantified self-reported activity as weekly minutes of MVPA to mirror our accelerometer-based analyses, which quantified total time spent performing activities of moderate or greater intensity.

### Additional exposures

Age, sex, body mass index (BMI), blood pressure, and anti-hypertensive medication use were assessed at the study visit most closely preceding accelerometer wear (accelerometer analysis) or IPAQ administration (self-reported activity analysis). Tobacco and alcohol use were obtained using standardized questionnaires. Alcohol use was quantified as total grams of alcohol consumed per week. The Townsend Deprivation Index was measured as a surrogate for socioeconomic deprivation^32^. As performed previously^33^, self-reported educational history and degree/qualification status were converted to years of educational attainment, and diet quality was classified as poor, intermediate, or good in accordance with responses to dietary questionnaries^34^. Individuals with missing BMI (0.2%) and blood pressure (0.1%) were excluded. For a minority of individuals (11%) who reported alcohol use frequency but not volume, we assumed the median volume observed at the reported frequency. A small number of individuals who did not report smoking status (0.002%) or medication use (0.4%) were assumed to be never smokers and not exposed to blood pressure medications, respectively. The sample median education level was assumed for individuals who did not report an education history (0.8%), and an intermediate diet quality for those who did not report sufficient dietary data (0.3%).

### Outcomes

We defined diseases using v1.2 of the Phecode Map^35^, a set of 1867 disease definitions arranged into clinically meaningful groups and identified using standardized sets of International Classification of Disease, 9th and 10th revision codes. Phecode definitions can be found at https://phewascatalog.org/. Diagnostic code sources included hospital data through linkage to national health-related datasets, as well as outpatient general practitioner visit data through linkage to electronic health records. Since general practitioner data are available only for a subset of UK Biobank participants (45%), we performed secondary analyses considering only hospital data. In our incident disease analyses of accelerometer-derived variables, person-time started at the end of accelerometer measurement and ended at an event, death, or last follow-up, whichever came first. In our incident disease analyses of self-reported activity, person-time started at enrollment and was otherwise constructed similarly. The date of last follow-up varied according to availability of linked health data and was therefore defined as March 31, 2021 for participants enrolled in England and Scotland, and February 28, 2018 for participants enrolled in Wales.

### Statistical analysis

Associations between accelerometer-derived MVPA (per 150 min/week, or approximately one standard deviation of the MVPA distribution) and incident disease were assessed using Cox proportional hazards regression. Individuals with prevalent disease at the time of exposure ascertainment were excluded from incident analyses of that disease. Given our aim to broadly identify plausible associations between MVPA and disease, and our intent to assess hundreds of disease outcomes simultaneously, we selected a uniform set of potential confounding variables to adjust for in our models. Specifically, we adjusted for age, sex, BMI, Townsend Deprivation Index, smoking status, alcohol use, anti-hypertensive medication use, systolic blood pressure, and diastolic blood pressure. Additional models were constructed using (a) adherence to standard physical activity guidelines (≥150 min of MVPA/week^1,10,11^) on the basis of accelerometer-derived MVPA, (b) overall mean acceleration, (c) self-reported MVPA, and (d) adherence to standard physical activity guidelines (≥150 min of MVPA/week^1,10,11^) on the basis of self-reported data, as secondary exposures of interest. To prevent model instability, only diseases with ≥120 events were tested (i.e., at least 10 events per variable in the primary model^36^). Given our interest in associations between physical activity and incident disease, as well as the age distribution of the sample, we excluded pregnancy conditions and congenital anomalies (*n* = 6 conditions after applying the minimum event filter), resulting in a total of 697 conditions tested in the primary analysis. To assess the robustness of potential associations to residual confounding, we report E-values^37^, which represent the minimum strength of association on the risk ratio scale that a potential confounder would need to have with the exposure and outcome to nullify the observed association. As suggested previously^37^, we present *E*-values for both the effect estimate and the limit of the confidence interval closer to the null.

To assess the ability of guideline-adherent activity to stratify risk of incident disease, we plotted the 5-year cumulative risk of heart failure, type 2 diabetes, cholelithiasis, and chronic bronchitis (exemplar conditions from each of the four disease categories having the greatest number of associations with physical activity), stratified at the guideline-based threshold of ≥150 min/week of MVPA. We also generated adjusted risk curves by plotting the predicted risk outputs from Cox models adjusted for each covariate included in the primary models, and stratified by guideline-adherent activity separately for men versus women. In these models, covariates were set to the sex-specific mean value (continuous variables), or most commonly observed value (categorical variables). To assess dose-response relationships between MVPA and disease risk, we plotted the relative hazard of incident disease for each quintile of MVPA, as compared to the lowest quintile, for each disease having a significant association with MVPA.

We performed several secondary analyses. First, we fit analogous models assessing for associations between measured MVPA and incident disease within age subgroups (i.e., <55, 55–64, and ≥65 years), approximating tertiles of the sample distribution. Second, we assessed associations with measured MVPA using alternative cutoffs of activity (i.e., ≥75 min and ≥300 min of MVPA/week), with the latter threshold representing activity levels recommended by the World Health Organization for additional health benefit^10^. Third, we performed analogous association testing between minutes of vigorous physical activity (mean acceleration >430 m*g*^19,29^) and disease. Fourth, to assess whether associations between activity and disease may have been driven by reverse causation, we repeated association testing of measured MVPA and incident disease in a landmark analysis in which person-time began two years after measured activity exposure. Fifth, we repeated association testing considering only hospital data (i.e., not general practitioner data) to define incident disease. Sixth, since BMI, systolic blood pressure, and diastolic blood pressure may serve as mediators of the effect of MVPA on diseases as opposed to confounders, we repeated association testing with these variables removed.

Except where otherwise specified, all analyses were performed using R v4.0^38^ (packages: ‘survival’, ‘data.table’, ‘fdrtool’). All *p* value thresholds were corrected for multiplicity by targeting a relatively stringent FDR threshold of 1%^39^. Tail-area based FDR thresholds were derived utilizing a generalized approach leveraging a modified Grenander distribution-based algorithm as described previously and implemented in R package ‘fdrtool’^40^. Directed acyclic graphs for the primary and secondary models are shown in Supplementary Fig. 20.

### Reporting summary

Further information on research design is available in the Nature Research Reporting Summary linked to this article.

## Supplementary information

Supplemental Material

Supplemental Data

Reporting Summary

## Data Availability

UK Biobank data are freely available for research purposes by application (https://www.ukbiobank.ac.uk/enable-your-research/register). Phecode-based outcomes developed for the current study will be returned to the UK Biobank for future research use within 6 months of publication.

## Code availability

Data processing scripts used to perform the analyses described herein can be found at (https://github.com/shaankhurshid/acceleration_phewas). Detailed summary data supporting major study results are provided in the Supplementary Data.
