# Supplementary material for: Wearable accelerometer-derived physical activity and incident disease
Source: NPJ Digit Med. 2022 Sep 2;5:131. doi: 10.1038/s41746-022-00676-9 (PMC9440134; doi:10.1038/s41746-022-00676-9)
Supplement: Supplementary file 1 — Supplemental Material [file 41746_2022_676_MOESM1_ESM.pdf]

Wearable Accelerometer-Derived Physical Activity and Incident Disease

Supplementary Material

Page	Item
1	**Supplementary Note.** Supplementary Data key
2	**Supplementary Figure 1.** Distribution of measured and self-reported MVPA
3	**Supplementary Figure 2.** Strongest associations between measured and self-reported MVPA and incident disease
4	**Supplementary Figure 3.** Total count of diseases within each category having significant associations with guideline-adherent physical activity
5	**Supplementary Figure 4.** Proportion of diseases within each category having significant associations with physical activity
6	**Supplementary Figure 5.** Associations between accelerometer-derived guideline-adherent physical activity and incident disease
7	**Supplementary Figure 6.** Multivariable-adjusted cumulative risk of disease stratified by level of physical activity in men
8	**Supplementary Figure 7.** Multivariable-adjusted cumulative risk of disease stratified by level of physical activity in women
9	**Supplementary Figure 8.** Moderate to vigorous activity levels associated with highest and lowest risks of disease
10	**Supplementary Figure 9.** Associations between measured MVPA and incident disease using varying thresholds
11	**Supplementary Figure 10.** Associations between self-reported MVPA and incident disease
12	**Supplementary Figure 11.** Associations between self-reported guideline-adherent activity and incident disease
13	**Supplementary Figure 12.** Associations between mean acceleration and incident disease
14	**Supplementary Figure 13.** Associations between vigorous activity and incident disease
15	**Supplementary Figure 14.** Vigorous activity levels associated with highest and lowest risks of disease
16	**Supplementary Figure 15.** Associations between quintile of measured vigorous physical activity and incident disease
17	**Supplementary Figure 16.** Associations between measured MVPA and incident disease across subgroups of age

Supplementary Figure 17. Associations between measured MVPA and incident disease using hospital data only 18

Supplementary Figure 18. Associations between measured MVPA and incident disease in models not adjusted for body mass index, blood pressure, or anti-hypetensive use 19

Supplementary Figure 19. Associations between measured MVPA and incident disease excluding events occurring within two years 20

Supplementary Figure 20. Directed acyclic graphs for primary and secondary models 21

Supplementary References 22

Supplementary Note. Supplementary Data key

1. accelerometer_mvpa **=** Associations with accelerometer-derived MVPA (per 1-standard deviation)
2. accelerometer_guideline = Associations with accelerometer-derived MVPA ≥150 minutes/week
3. accelerometer_cutoff_75min = Associations with accelerometer-derived MVPA ≥75 minutes/week
4. accelerometer_cutoff_300min = Associations with accelerometer-derived MVPA ≥300 minutes/week
5. mean_acceleration = Associations with mean acceleration (per 1-standard deviation)
6. self_mvpa **=** Associations with self-reported MVPA (per 1-standard deviation)
7. self_guideline = Associations with self-reported MVPA ≥150 minutes/week
8. vigorous_activity = Associations with accelerometer-derived vigorous physical activity (per 1-standard deviation)
9. accelerometer_mvpa_quintile = Associations with accelerometer-derived MVPA per quintile (1st quintile referent)
10. accelerometer_vigorous_quintile = Associations with accelerometer-derived vigorous physical activity per quintile (1st quintile referent)
11. accelerometer_mvpa_2yr_blanked = Associations with accelerometer-derived MVPA (per 1-standard deviation) excluding events within 2 years of accelerometer wear
12. accelerometer_mvpa_inpt_only = Associations with accelerometer-derived MVPA (per 1-standard deviation) with outcomes defined using hopsital data only
13. accelerometer_mvpa_less_55 = Associations with accelerometer-derived MVPA (per 1-standard deviation) among individuals aged <55 years
14. accelerometer_mvpa_less_55 = Associations with accelerometer-derived MVPA (per 1-standard deviation) among individuals aged 55-64 years
15. accelerometer_mvpa_less_55 = Associations with accelerometer-derived MVPA (per 1-standard deviation) among individuals aged ≥65 years
16. accelerometer_mvpa_secondary_mod = Associations with accelerometer-derived MVPA (per 1-standard deviation) in models not adjusted for body mass index, systolic blood pressure, diastolic blood pressure, or anti-hypertensive use

Supplementary Figure 1. Distribution of measured and self-reported MVPA

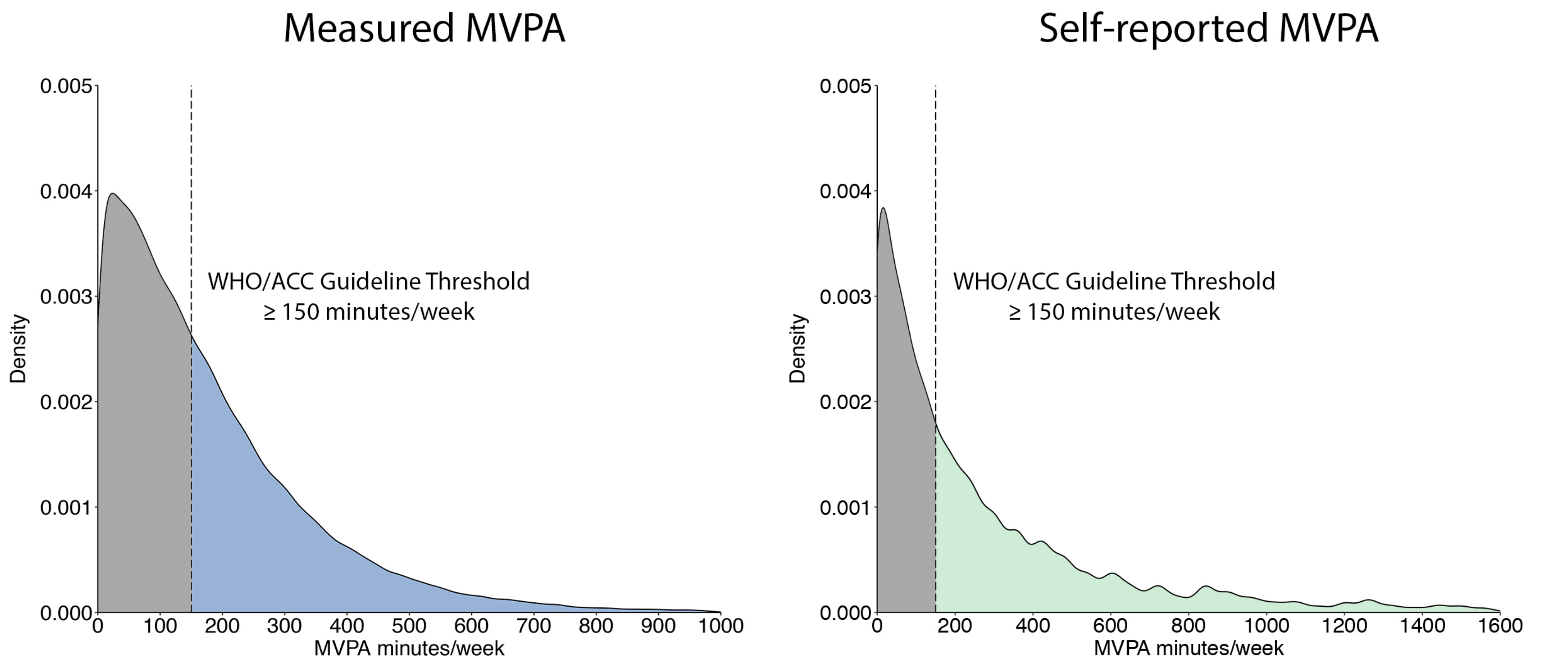

Depicted are the distributions of accelerometer-derived moderate-to-vigorous physical activity (MVPA, left) and self-reported MVPA (right). The left plot represents the accelerometer sample (N=96,244) and the right plot represents the self-reported activity sample (N=456,374). In both plots, the hashed vertical line represents the level of MVPA recommended by consensus activity guidelines (i.e., ≥150 minutes/week[1–3]), and the grayed area represents individuals whose activity is below the threshold. Values above the 95th percentile may be truncated for graphical purposes.

Supplementary Figure 2. Strongest associations between measured and self-reported MVPA and incident disease

a

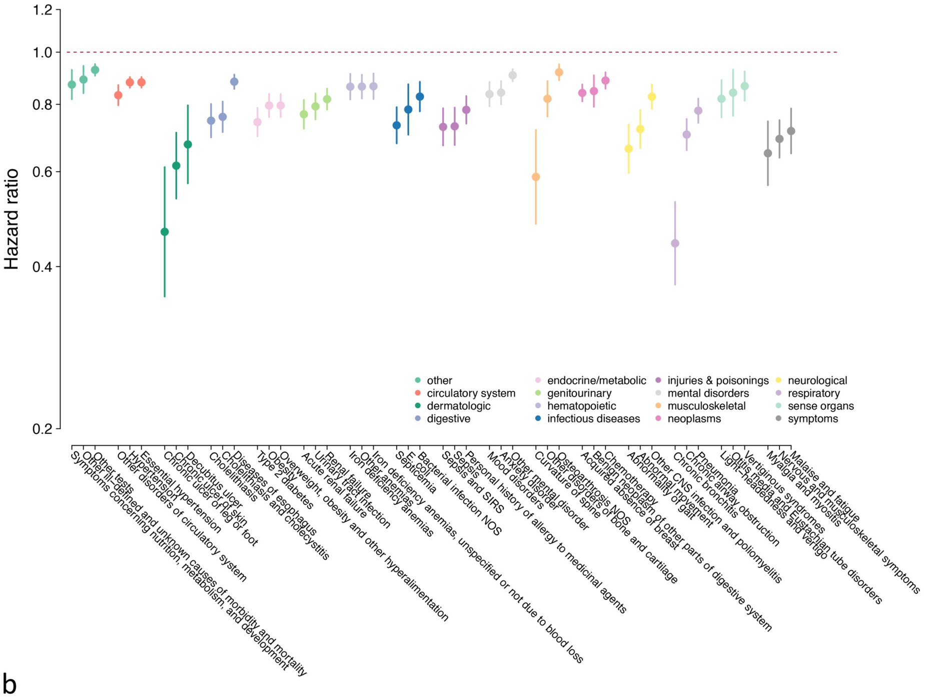

b

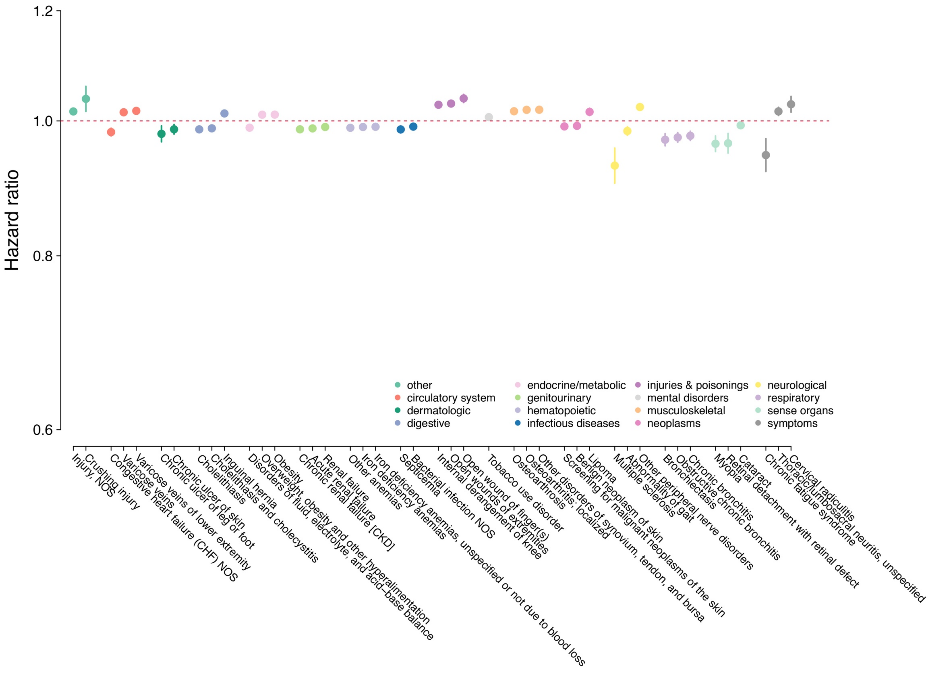

Panel a depicts the top three conditions in each disease category having the strongest associations (i.e., smallest p-values) with accelerometer-derived moderate-vigorous physical activity (MVPA). **Panel b** depicts the top three conditions in each disease category having the strongest associations with self-reported MVPA. Diseases are colored by category (see legends), and hazard ratios are per 150-minute increase in weekly MVPA.

Supplementary Figure 3. Total count of diseases within each category having significant associations with moderate to vigorous physical activity

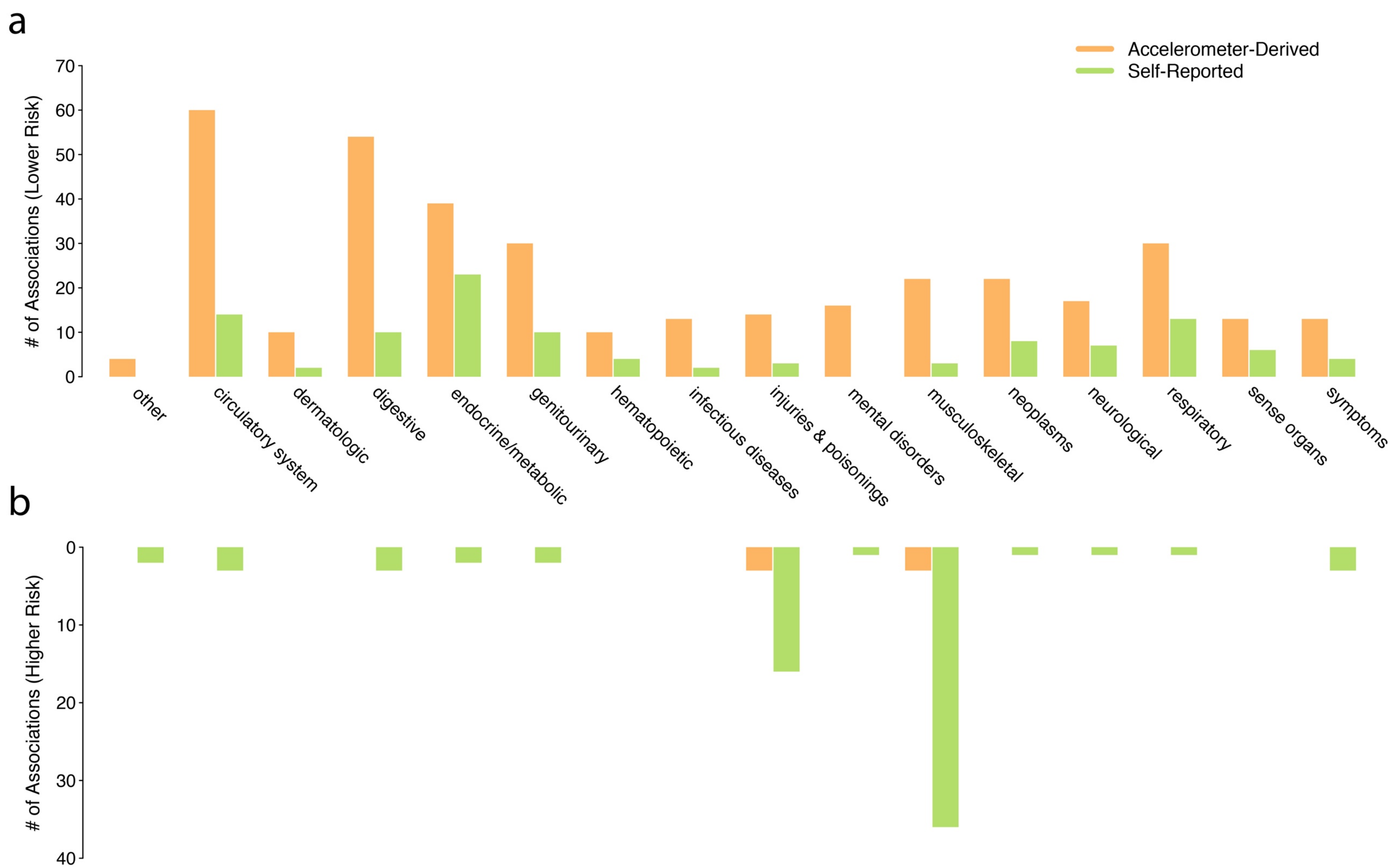

Depicted is the total count of diseases within each category having a significant association with moderate to vigorous physical activity (MVPA). Accelerometer measured and self-reported MVPA are compared (see legend). The top panel depicts associations indicating lower disease risk (i.e., hazard ratios less than one), while the bottom panel depicts associations indicating higher disease risk (i.e., hazard ratios greater than one). Only associations significant at a false discovery rate of 1% are depicted.

Supplementary Figure 4. Proportion of diseases within each category having significant associations with moderate to vigorous physical activity

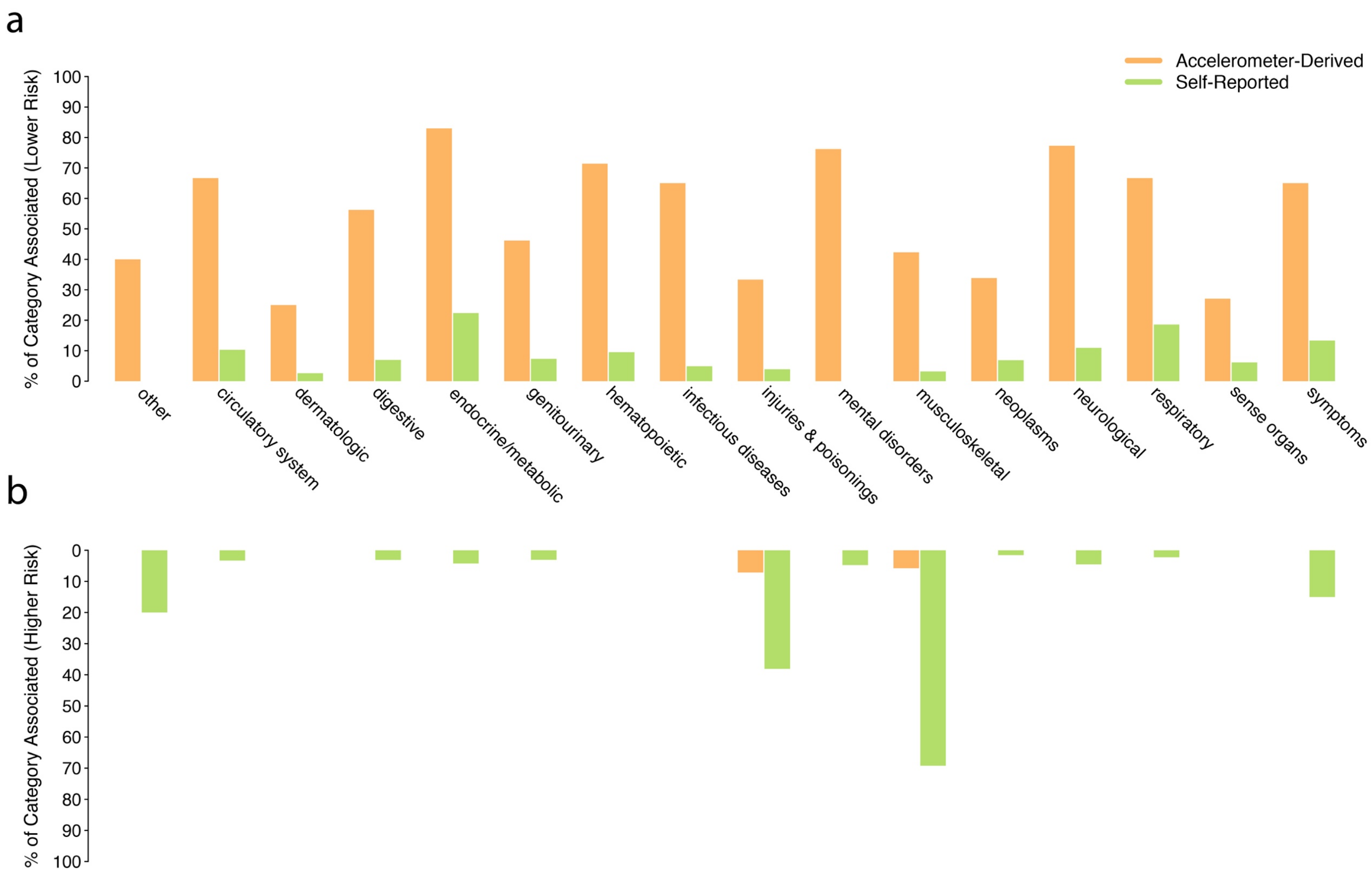

Depicted is the proportion of diseases within each category having a significant association with moderate to vigorous physical activity (MVPA). Accelerometer measured and self-reported MVPA are compared (see legend). The top panel depicts associations indicating lower disease risk (i.e., hazard ratios less than one), while the bottom panel depicts associations indicating higher disease risk (i.e., hazard ratios greater than one). Only associations significant at a false discovery rate of 1% are depicted.

Supplementary Figure 5. Associations between accelerometer-derived guideline-adherent physical activity and incident disease

a

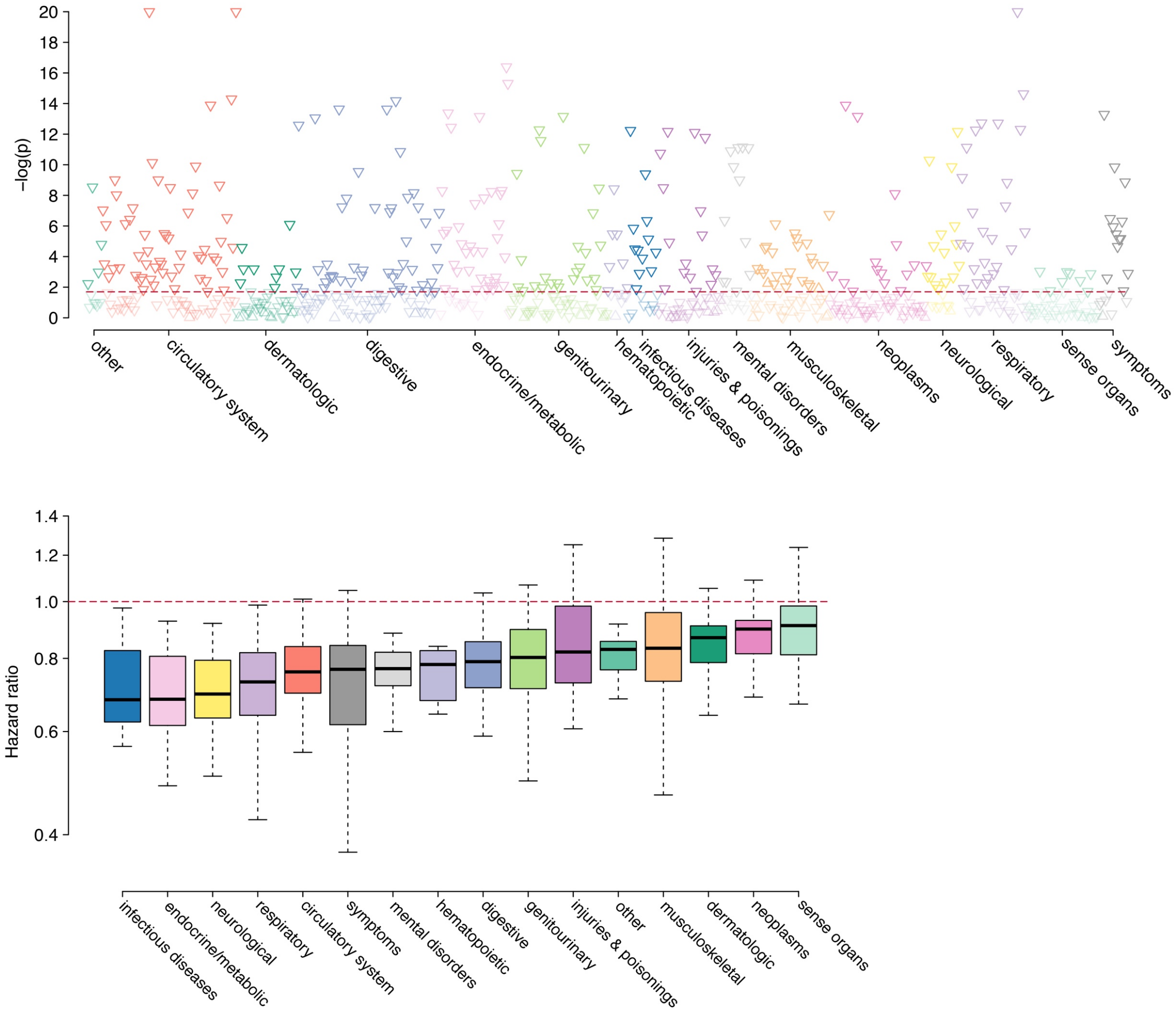

Depicted are the results of phenome-wide disease association testing with accelerometer-derived activity meeting guideline recommendations (≥150 minutes of moderate-vigorous physical activity per week[1–3]) as the exposure of interest, in Cox proportional hazards models adjusted for multiple covariates (see text). **Panel a** plots the negative log10 p-value for the association between guideline-adherent activity and each individual disease (grouped by category on the x-axis), with darker shaded points meeting significance at a false discovery rate of 1% (threshold depicted by horizontal dashed red line). Upward facing triangles represent higher risk (hazard ratios > 1), while downward facing triangles represent lower risk (hazard ratio < 1). P-values smaller than $1x10^{-20}$ are displayed as $1x10^{-20}$ for graphical purposes. **Panel b** shows the distribution of hazard ratios observed in the presence of guideline-adherent activity across each disease category (x-axis), with the center line depicting the within-category median hazard ratio, the bounds of the box representing quartile 1 to quartile 3, and the whiskers extending 1.5 interquartile ranges beyond the box. Categories are arranged by increasing median hazard ratio, from lowest (left) to highest (right).

Supplementary Figure 6. Multivariable-adjusted cumulative risk of disease stratified by level of physical activity in men

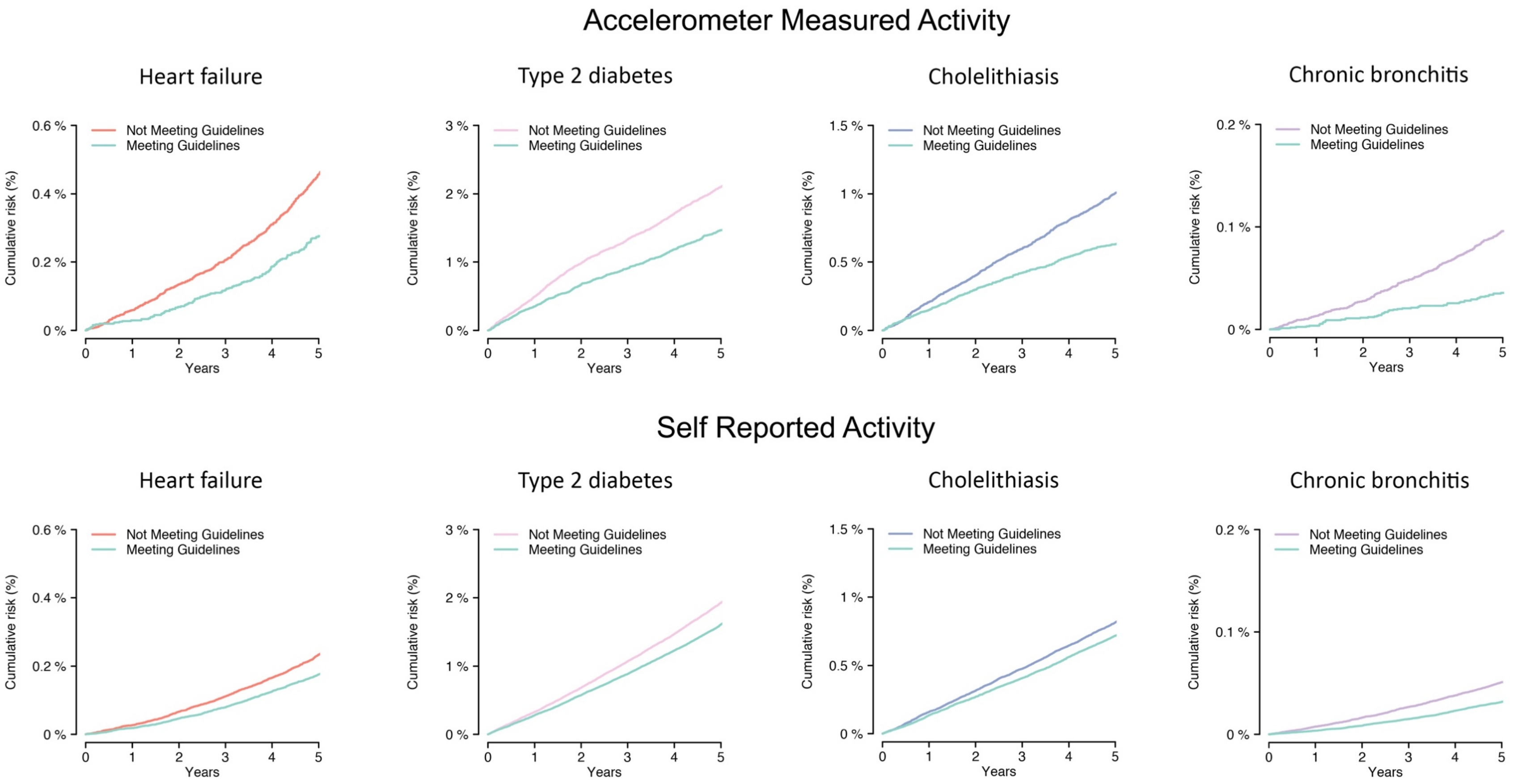

Depicted is the adjusted 5-year cumulative risk of heart failure, type 2 diabetes, cholelithiasis, and chronic bronchitis for average risk men, stratified by guideline-adherent activity (≥150 minutes of MVPA/week[1–3]) according to accelerometer-derived MVPA (top panels) and according to self-reported MVPA (bottom). Curves are derived from stratified Cox models with each respective disease as the outcome and guideline-adherent activity as a stratification variable. All covariates included in the primary model were included as covariates, with the sex-specific mean value (continuous variables), or most commonly observed value (categorical variables) assumed. Representative diseases were selected from the four categories having the greatest enrichment for associations with activity, where each disease was significantly associated with both accelerometer-derived and self-reported activity at a false discovery rate of 1%.

Supplementary Figure 7. Multivariable-adjusted cumulative risk of disease stratified by level of physical activity in women

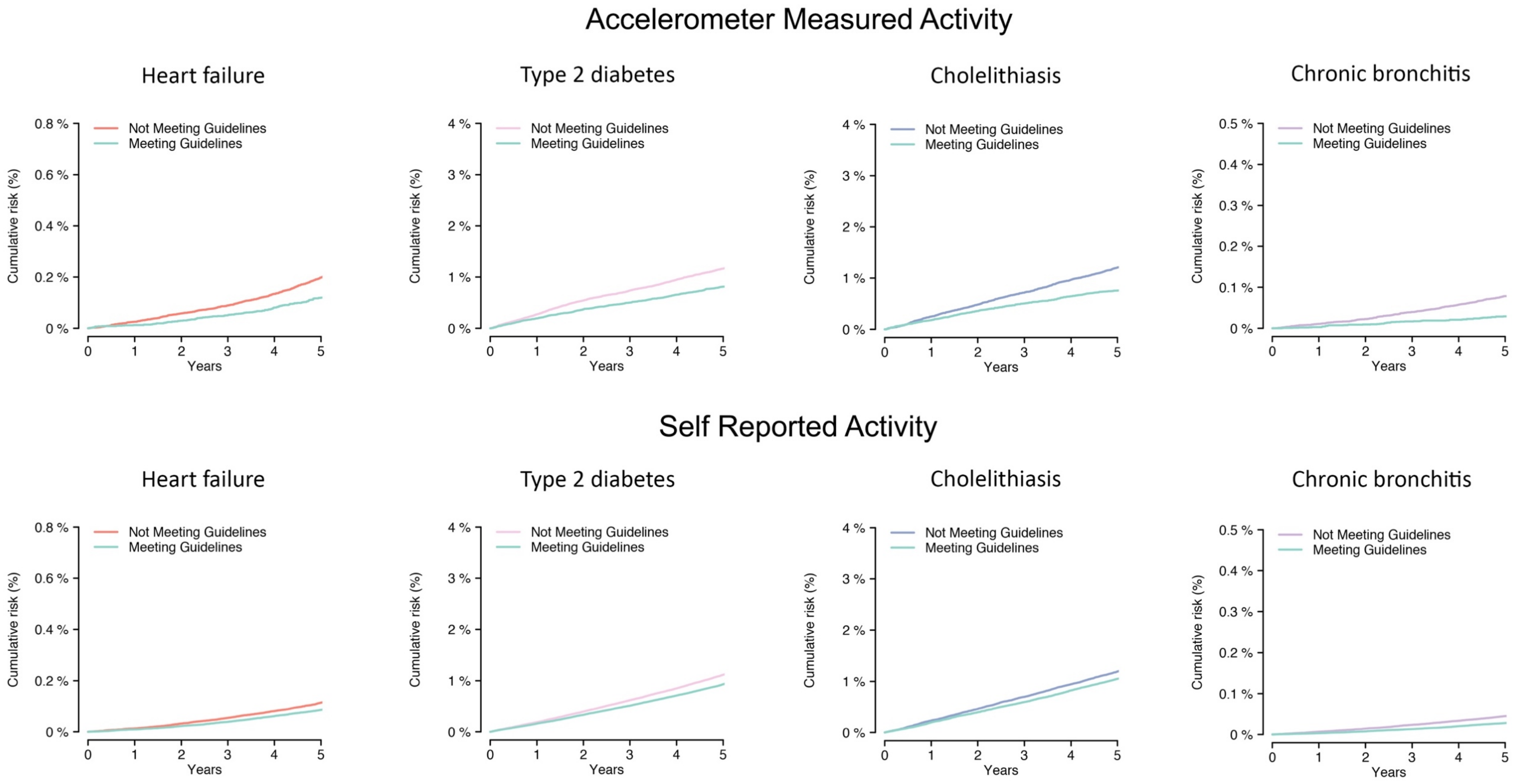

Depicted is the adjusted 5-year cumulative risk of heart failure, type 2 diabetes, cholelithiasis, and chronic bronchitis for average risk women, stratified by guideline-adherent activity (≥150 minutes of MVPA/week[1–3]) according to accelerometer-derived MVPA (top panels) and according to self-reported MVPA (bottom). Curves are derived from stratified Cox models with each respective disease as the outcome and guideline-adherent activity as a stratification variable. All covariates included in the primary model were included as covariates, with the sex-specific mean value (continuous variables), or most commonly observed value (categorical variables) assumed. Representative diseases were selected from the four categories having the greatest enrichment for associations with activity, where each disease was significantly associated with both accelerometer-derived and self-reported activity at a false discovery rate of 1%.

Supplementary Figure 8. Moderate to vigorous activity levels associated with highest and lowest risks of disease

a

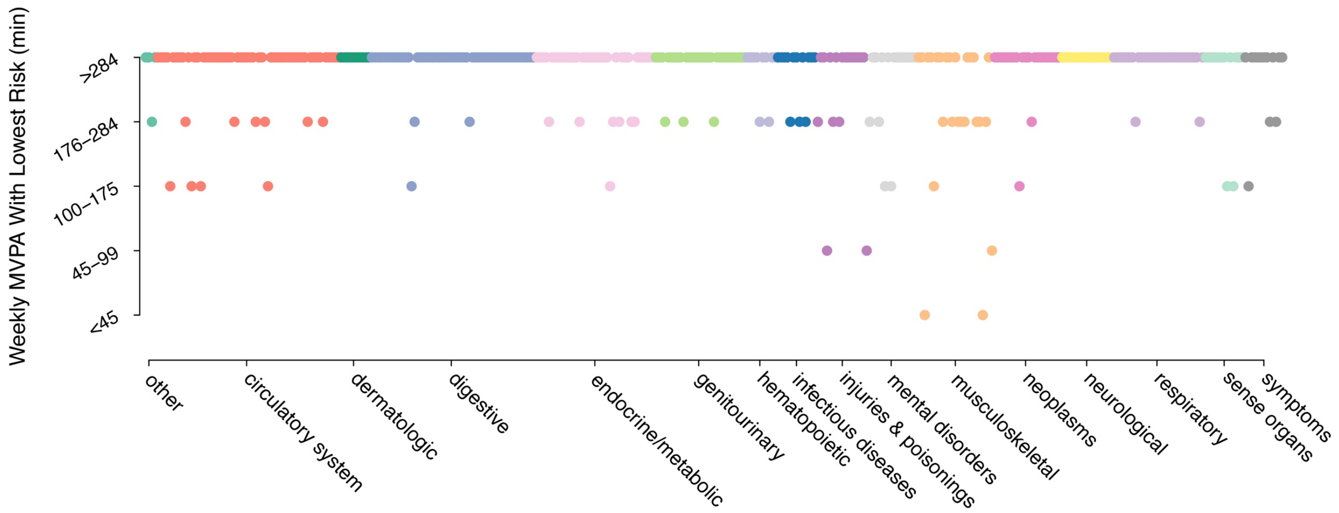

b

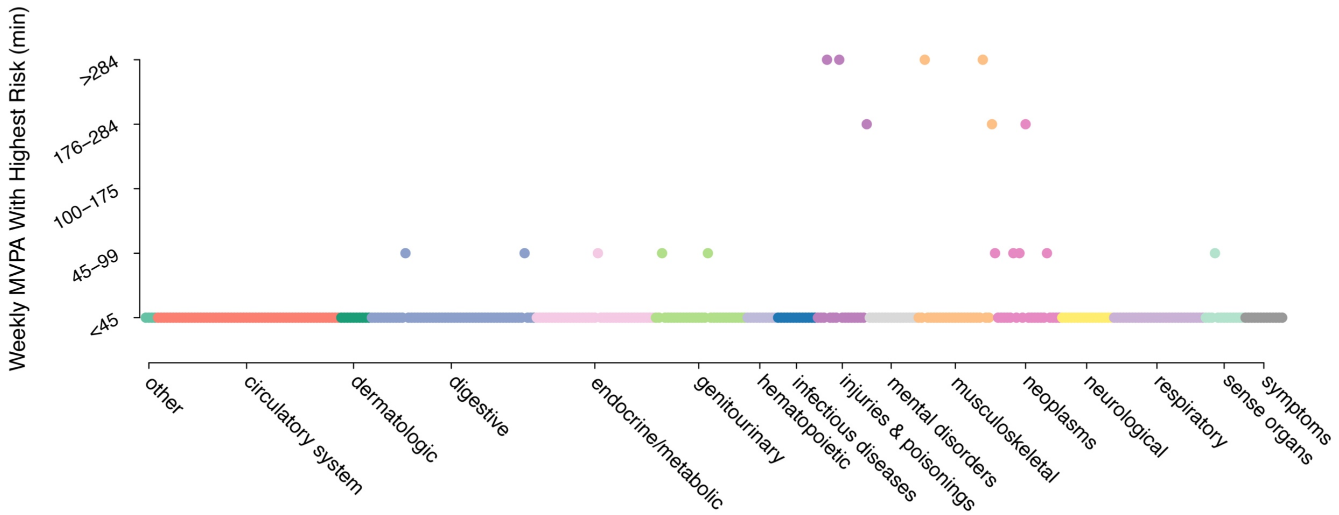

Depicted are the moderate to vigorous physical activity (MVPA) quintiles (values indicated on y-axis) associated with the lowest risk of incident disease (**panel a**, top), and those associated with the highest risk of incident disease (**panel b**, bottom), across disease category (x-axis). Each point depicts the relevant quintile for an individual disease. Values were derived from multivariable adjusted Cox proportional hazards models utilizing quintile of MVPA as the exposure of interest, with the highest and lowest quintiles corresponding to the highest and lowest absolute predicted disease risk according to the model (see text). Only diseases having significant associations with MVPA at the FDR threshold of 1% are depicted.

Supplementary Figure 9. Associations between measured MVPA and incident disease using varying thresholds

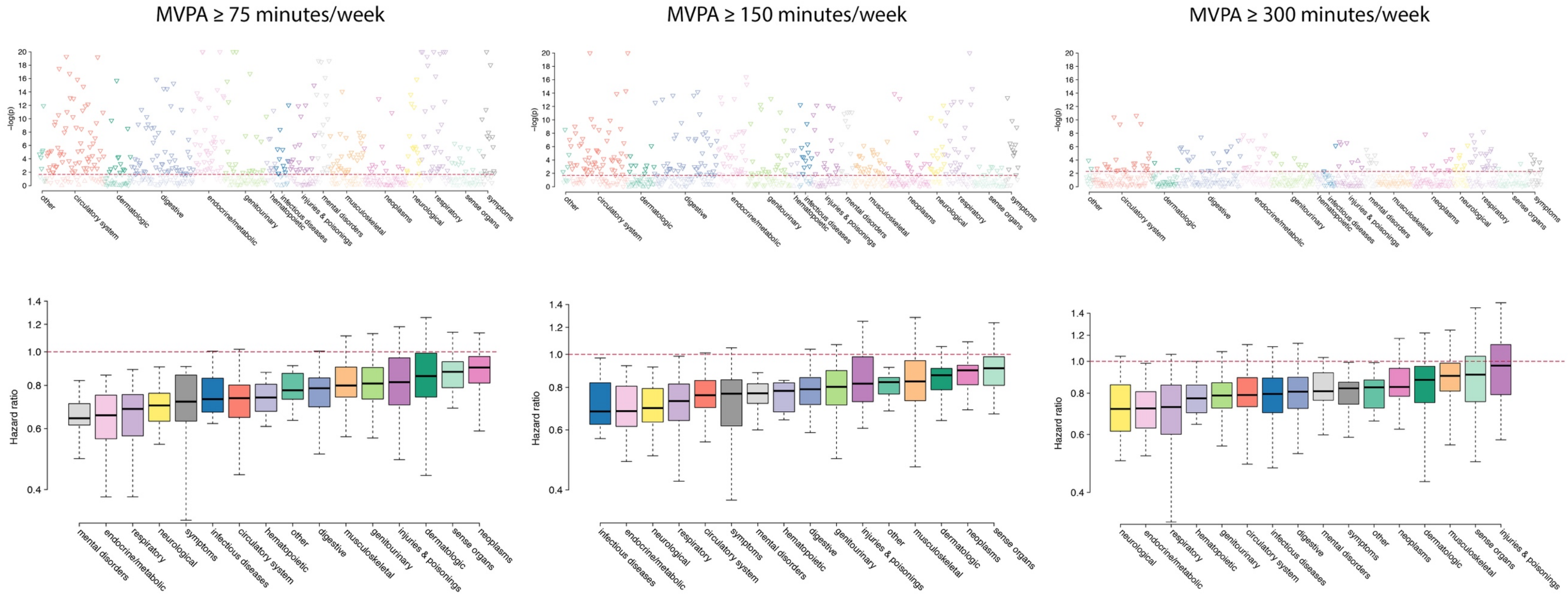

Depicted are associations between measured moderate-vigorous physical activity (MVPA) and incident disease applying varying thresholds. Middle panels depict the standard guideline-based threshold used in the primary analysis (≥150 minutes MVPA/week). Right panels depict the World Health Organization threshold for extended health benefit (≥300 minutes MVPA/week).[3] The left panels depict a lower threshold (≥75 minutes MVPA/week). The number of individuals meeting each threshold are as follows: ≥75 minutes MVPA/week: 67,006 (69.6%), ≥150 minutes MVPA/week: 44,603 (46.3%), ≥300 minutes MVPA/week: 17,832 (18.5%). Upper panels depict the negative log10 p-value for the association between activity meeting the given threshold and each individual disease (grouped by category on the x-axis), with darker shaded points meeting significance at a false discovery rate of 1% (threshold depicted by horizontal dashed red line). P-values smaller than $1x10^{-20}$ are displayed as $1x10^{-20}$ for graphical purposes. Upward facing triangles represent higher risk (hazard ratios > 1), while downward facing triangles represent lower risk (hazard ratio < 1). Lower panels depict the distribution of hazard ratios observed across each disease category (x-axis), with the center line depicting the within-category median hazard ratio, the bounds of the box representing quartile 1 to quartile 3, and the whiskers extending 1.5 interquartile ranges beyond the box. Categories are arranged by increasing median hazard ratio, from lowest (left) to highest (right).

Supplementary Figure 10. Associations between self-reported MVPA and incident disease

a

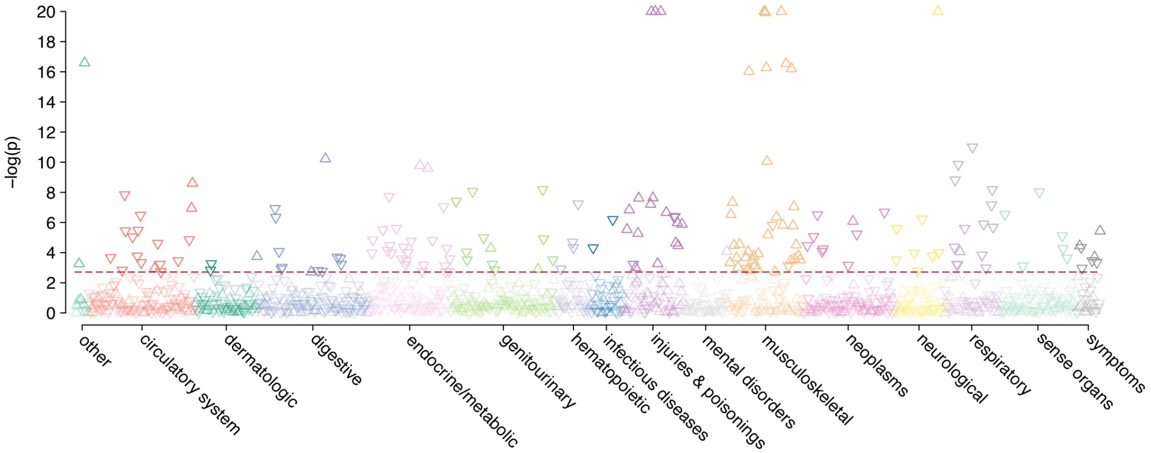

b

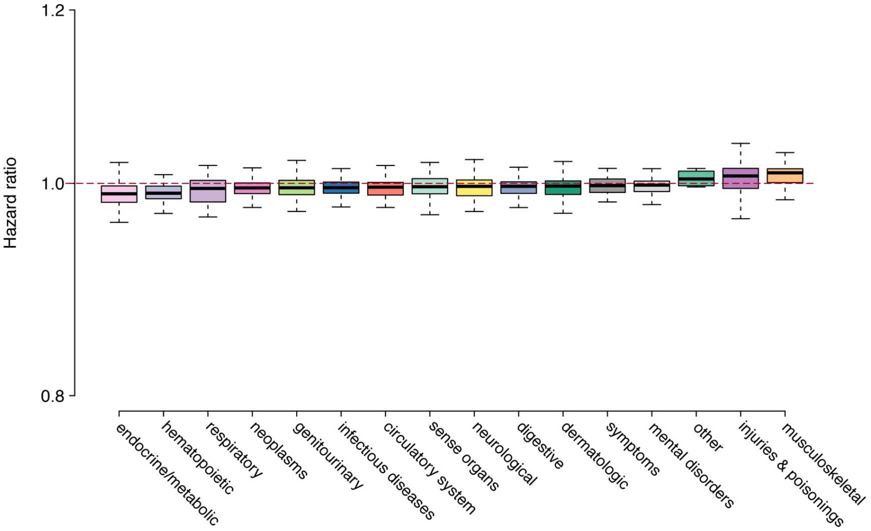

Depicted are the results of phenome-wide disease association testing with self-reported moderate-to-vigorous physical activity (MVPA) as the exposure of interest, in multivariable adjusted Cox proportional hazards models. **Panel a** plots the negative log10 p-value for the association between self-reported MVPA and each individual disease (grouped by category on the x-axis), with darker shaded points meeting significance at a false discovery rate of 1% (threshold depicted by horizontal dashed red line). Upward facing triangles represent higher risk (hazard ratios > 1), while downward facing triangles represent lower risk (hazard ratio < 1). P-values smaller than $1x10^{-20}$ are displayed as $1x10^{-20}$ for graphical purposes. **Panel b** shows the distribution of hazard ratios observed for every 150-minute increase in self-reported weekly MVPA across each disease category (x-axis), with the center line depicting the within-category median hazard ratio, the bounds of the box representing quartile 1 to quartile 3, and the whiskers extending 1.5 interquartile ranges beyond the box. Categories are arranged by increasing median hazard ratio, from lowest (left) to highest (right).

Supplementary Figure 11. Associations between self-reported guideline-adherent activity and incident disease

a

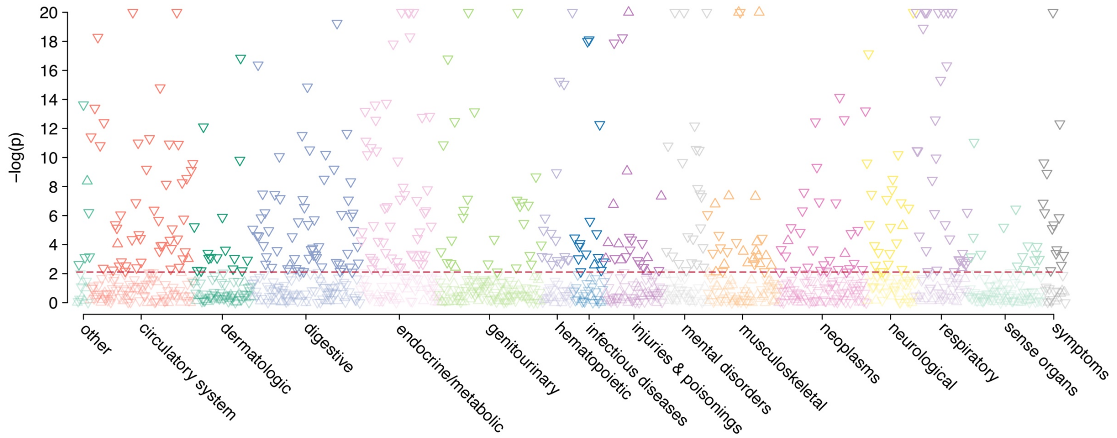

b

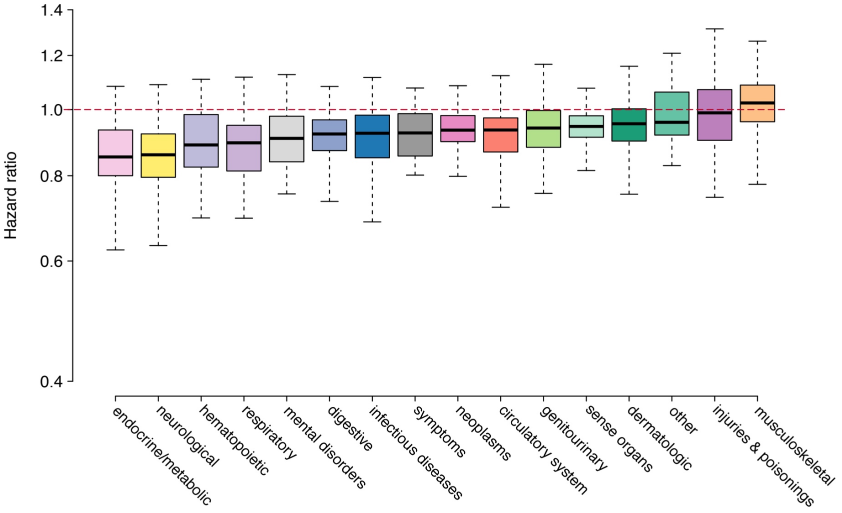

Depicted are the results of phenome-wide disease association testing with self-reported activity meeting guideline recommendations (≥150 minutes of moderate-vigorous physical activity per week[1–3]) as the exposure of interest, in multivariable adjusted Cox proportional hazards models. **Panel a** plots the negative log10 p-value for the association between self-reported guideline-adherent activity and each individual disease (grouped by category on the x-axis), with darker shaded points meeting significance at a false discovery rate of 1% (threshold depicted by horizontal dashed red line). Upward facing triangles represent higher risk (hazard ratios > 1), while downward facing triangles represent lower risk (hazard ratio < 1). P-values smaller than $1x10^{-20}$ are displayed as $1x10^{-20}$ for graphical purposes. **Panel b** shows the distribution of hazard ratios observed in the presence of guideline-adherent activity across each disease category (x-axis), with the center line depicting the within-category median hazard ratio, the bounds of the box representing quartile 1 to quartile 3, and the whiskers extending 1.5 interquartile ranges beyond the box. Categories are arranged by increasing median hazard ratio, from lowest (left) to highest (right).

Supplementary Figure 12. Associations between mean acceleration and incident disease

a

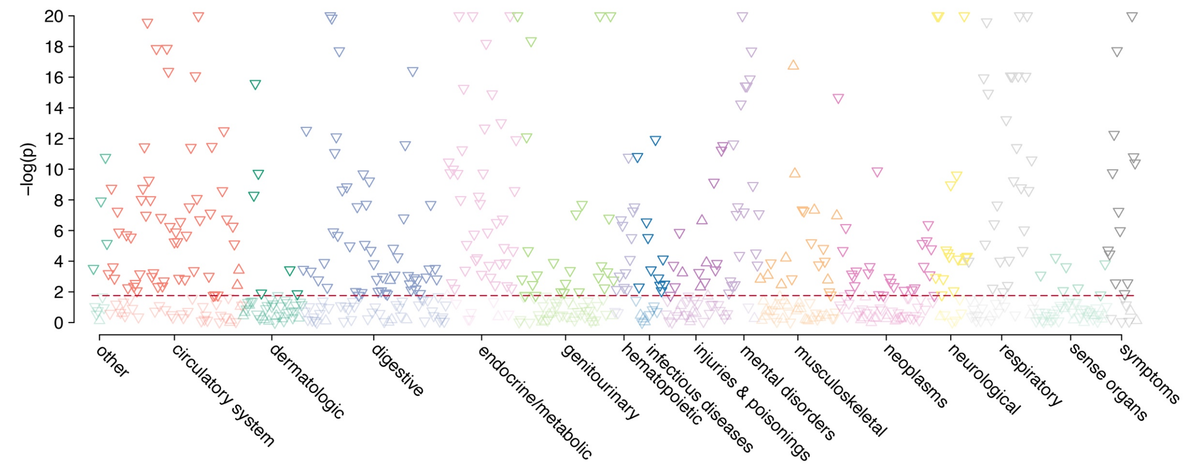

b

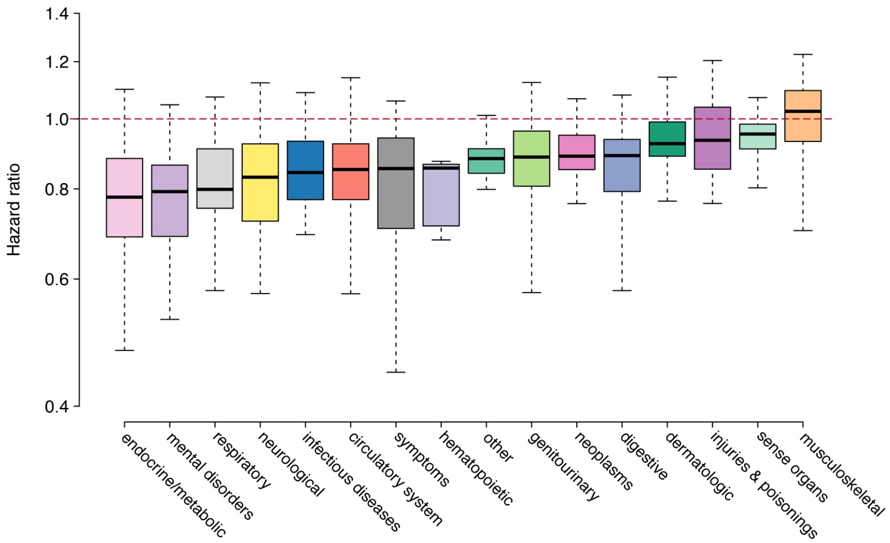

Depicted are the results of phenome-wide disease association testing with mean acceleration as the exposure of interest, in Cox proportional hazards models adjusted for age, sex, and body mass index. **Panel a** plots the negative log10 p-value for the association between mean acceleration and each individual disease (grouped by category on the x-axis), with darker shaded points meeting significance at a false discovery rate of 1% (threshold depicted by horizontal dashed red line). Upward facing triangles represent higher risk (hazard ratios > 1), while downward facing triangles represent lower risk (hazard ratio < 1). P-values smaller than $1x10^{-20}$ are displayed as $1x10^{-20}$ for graphical purposes. **Panel b** shows the distribution of hazard ratios observed per 1 standard deviation increase in mean acceleration across each disease category (x-axis), with the center line depicting the within-category median hazard ratio, the bounds of the box representing quartile 1 to quartile 3, and the whiskers extending 1.5 interquartile ranges beyond the box. Categories are arranged by increasing median hazard ratio, from lowest (left) to highest (right).

Supplementary Figure 13. Associations between vigorous activity and incident disease

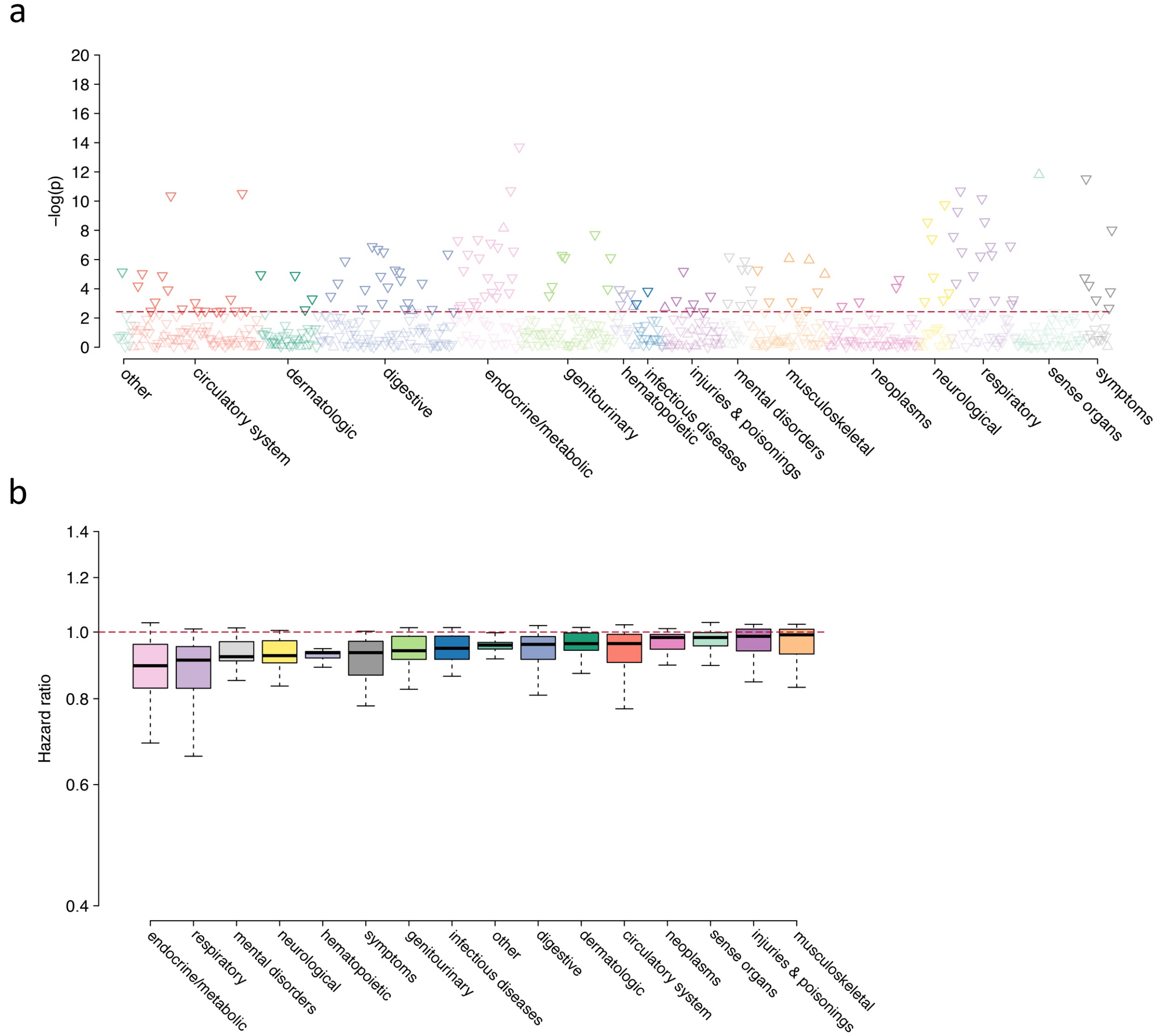

Depicted are the results of phenome-wide disease association testing with minutes of vigorous physical activity (i.e., mean acceleration >430m*g*[4,5]) as the exposure of interest, in multivariable adjusted Cox proportional hazards models. **Panel a** plots the negative log10 p-value for the association between vigorous activity and each individual disease (grouped by category on the x-axis), with darker shaded points meeting significance at a false discovery rate of 1% (threshold depicted by horizontal dashed red line). Upward facing triangles represent higher risk (hazard ratios > 1), while downward facing triangles represent lower risk (hazard ratio < 1). **Panel b** shows the distribution of hazard ratios observed per 150-minute increase in weekly vigorous activity across each disease category (x-axis), with the center line depicting the within-category median hazard ratio, the bounds of the box representing quartile 1 to quartile 3, and the whiskers extending 1.5 interquartile ranges beyond the box. Categories are arranged by increasing median hazard ratio, from lowest (left) to highest (right).

Supplementary Figure 14. Vigorous activity levels associated with highest and lowest risks of disease

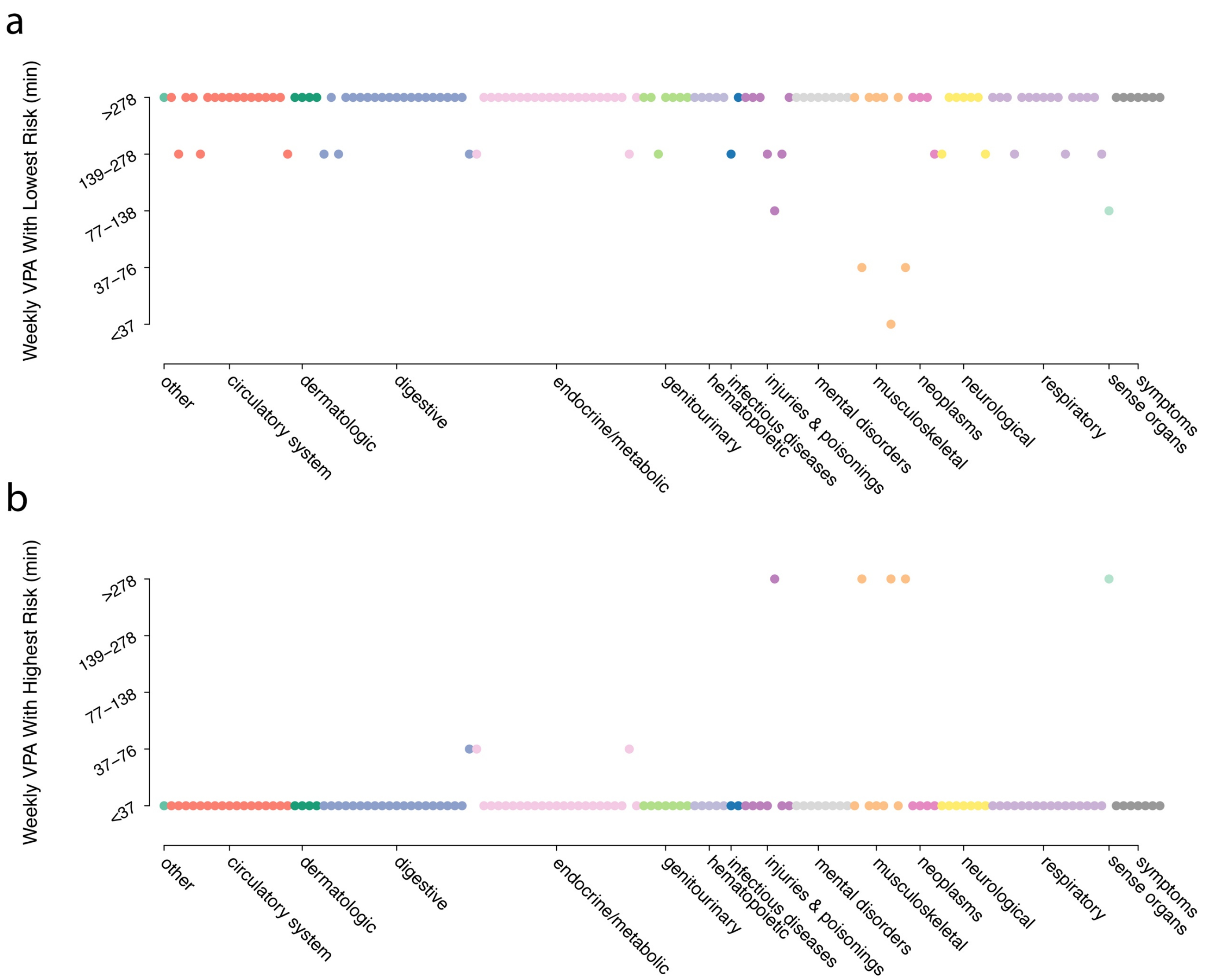

Depicted are the vigorous physical activity quintiles (values indicated on y-axis) associated with the lowest risk of incident disease (**panel a**, top), and those associated with the highest risk of incident disease (**panel b**, bottom), across disease category (x-axis). Each point depicts the relevant quintile for an individual disease. Values were derived from multivariable adjusted Cox proportional hazards models utilizing quintile of vigorous physical activity as the exposure of interest, with the highest and lowest quintiles corresponding to the highest and lowest absolute predicted disease risk according to the model (see text). Only diseases having significant associations with vigorous activity at the FDR threshold of 1% are depicted.

Supplementary Figure 15. Associations between quintile of measured VPA and incident disease

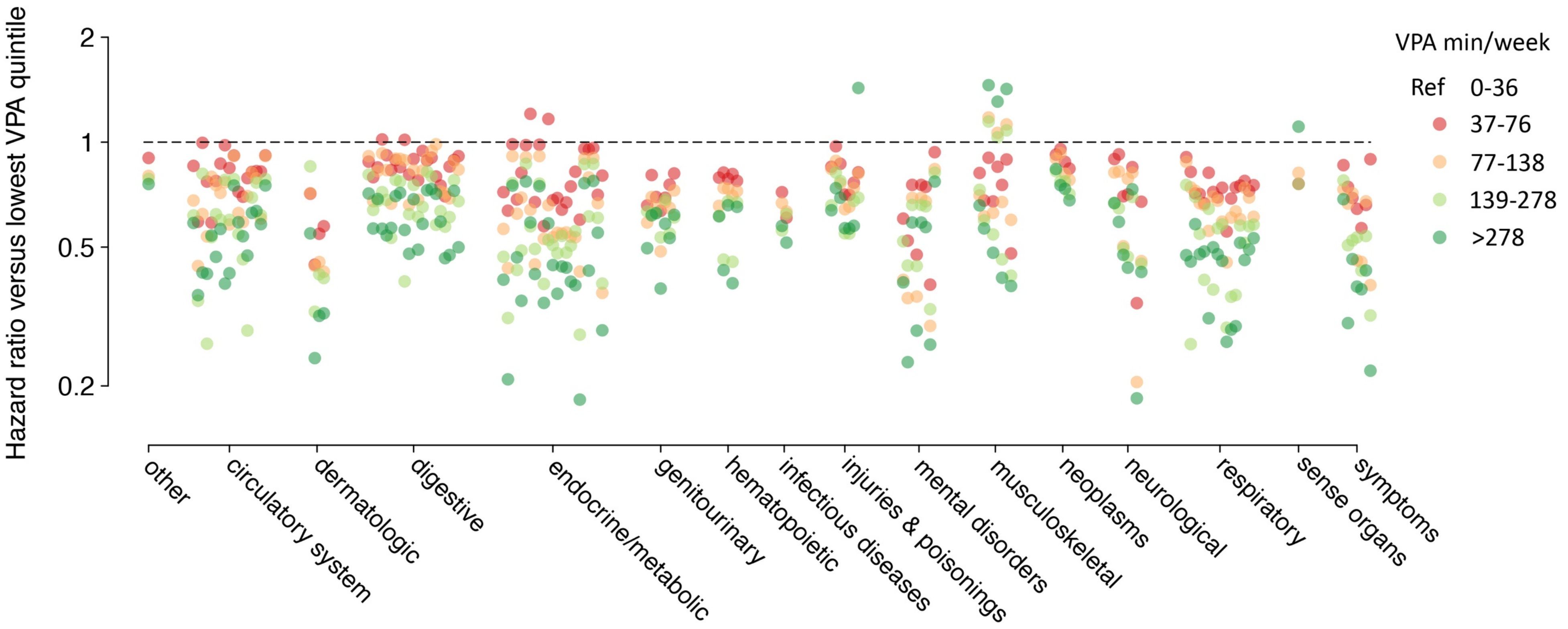

Depicted is the relative hazard of incident disease according to quintile of VPA and grouped by category. Each disease is represented by four points (see legend), with each point representing the hazard ratio associated with a given quintile of VPA, as compared to the lowest quintile as the referent. VPA volumes corresponding to each quintile are shown in the legend. The hashed horizontal line depicts a hazard ratio of one (i.e., equal hazard to the lowest quintile reference group).

Supplementary Figure 16. Associations between measured MVPA and incident disease across subgroups of age

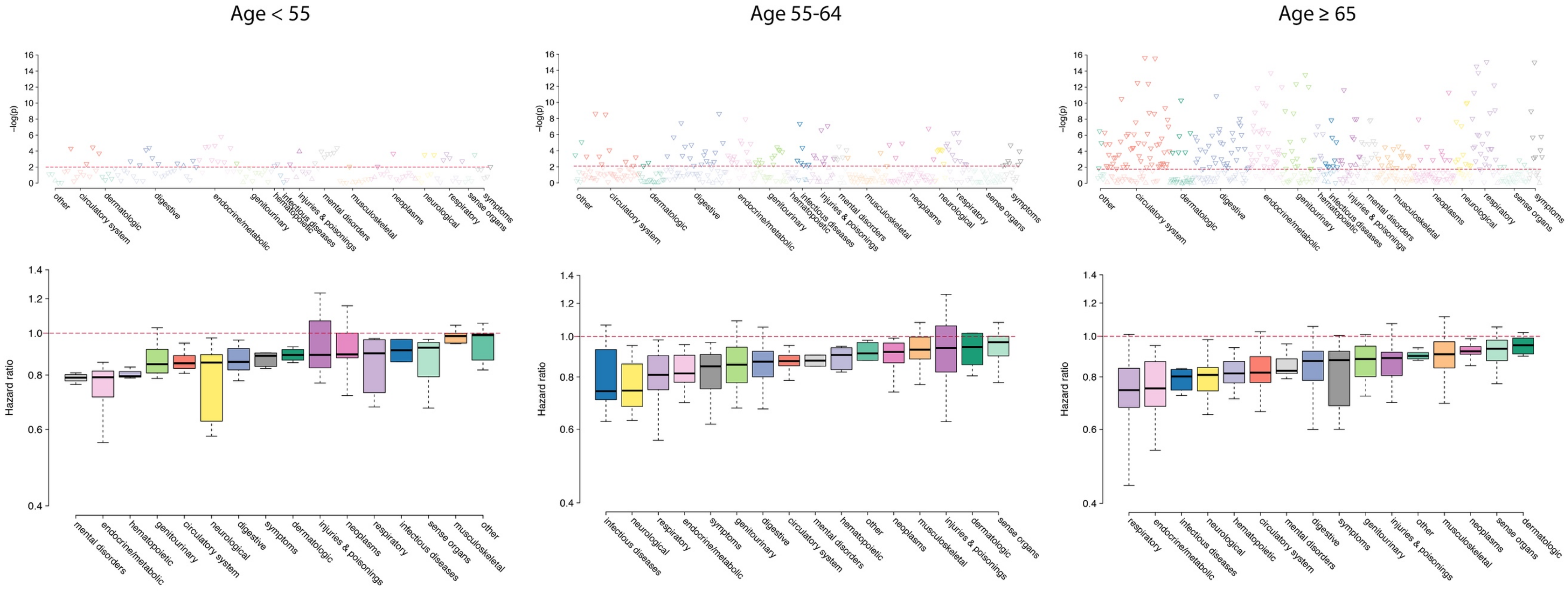

Depicted are associations between accelerometer-derived moderate-vigorous physical activity (MVPA) and incident disease across subgroups of age. Age cutoffs were chosen to approximate tertiles of the sample distribution (subgroup n for age <55: 20,505, age 55-65: 33,811, age ≥ 65: 41,928). Upper panels depict the negative log10 p-value for the association between MVPA and each individual disease (grouped by category on the x-axis), with darker shaded points meeting significance at a false discovery rate of 1% (threshold depicted by horizontal dashed red line). Upward facing triangles represent higher risk (hazard ratios > 1), while downward facing triangles represent lower risk (hazard ratio < 1). Lower panels depict the distribution of hazard ratios observed per 150-minute increase in weekly MVPA across each disease category (x-axis), with the center line depicting the within-category median hazard ratio, the bounds of the box representing quartile 1 to quartile 3, and the whiskers extending 1.5 interquartile ranges beyond the box. Categories are arranged by increasing median hazard ratio, from lowest (left) to highest (right).

Supplementary Figure 17. Associations between measured MVPA and incident disease classified using hospital data only

a

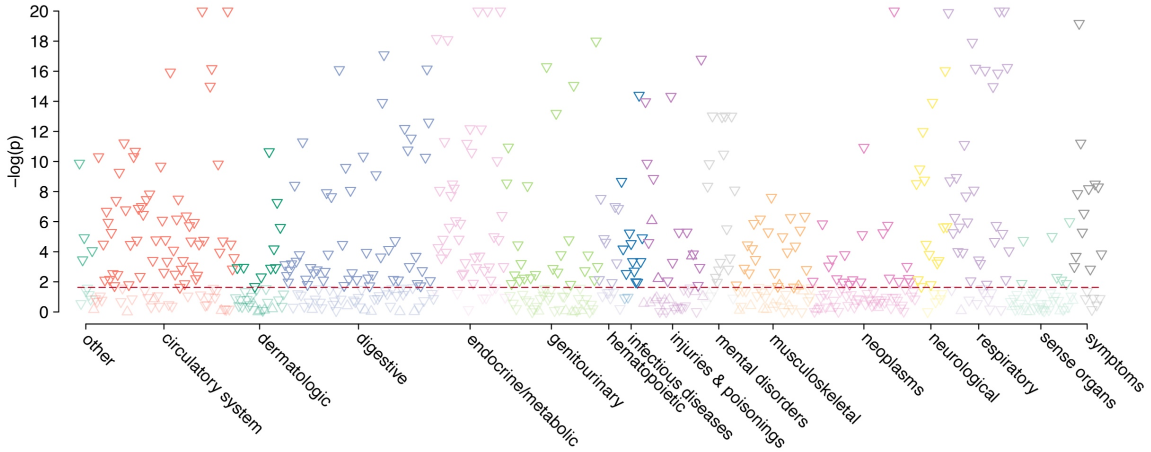

b

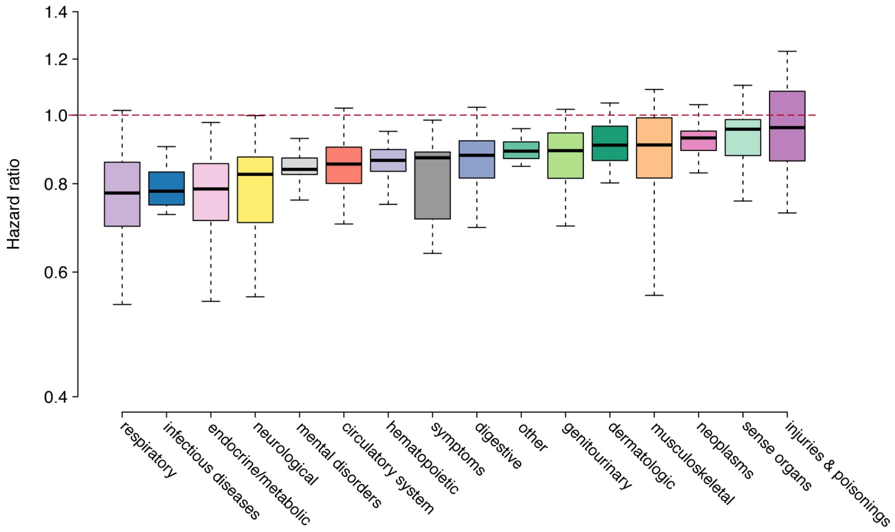

Depicted are the results of phenome-wide disease association testing with accelerometer measured moderate-to-vigorous physical activity (MVPA) as the exposure of interest, in multivariable adjusted Cox proportional hazards models. Outcomes were defined using hospital data only (i.e., not considering general practitioner data). **Panel a** plots the negative log10 p-value for the association between measured MVPA and each individual disease (grouped by category on the x-axis), with darker shaded points meeting significance at a false discovery rate of 1% (threshold depicted by horizontal dashed red line). Upward facing triangles represent higher risk (hazard ratios > 1), while downward facing triangles represent lower risk (hazard ratio < 1). **Panel b** shows the distribution of hazard ratios observed per 150-minute increase in weekly MVPA across each disease category (x-axis), with the center line depicting the within-category median hazard ratio, the bounds of the box representing quartile 1 to quartile 3, and the whiskers extending 1.5 interquartile ranges beyond the box. Categories are arranged by increasing median hazard ratio, from lowest (left) to highest (right).

Supplementary Figure 18. Associations between measured MVPA and incident disease in models not adjusted for body mass index, blood pressure, or anti-hypertensive use

a

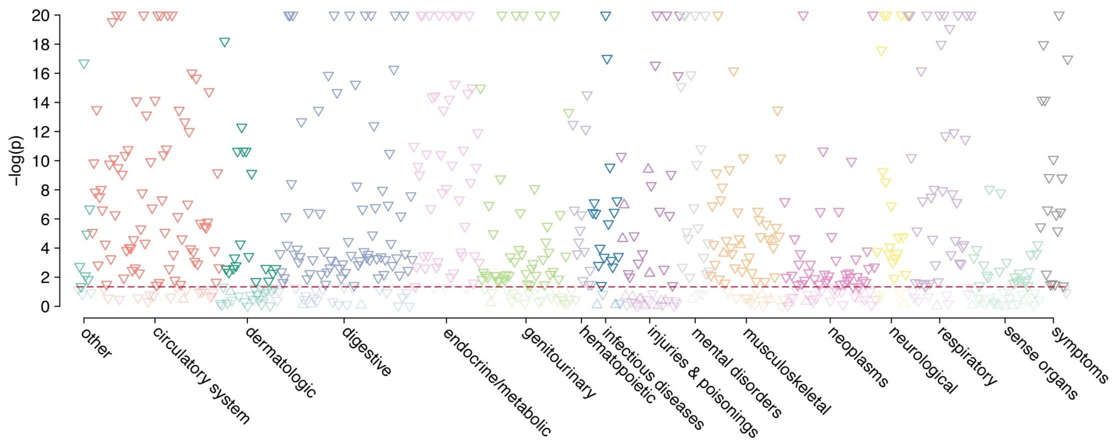

b

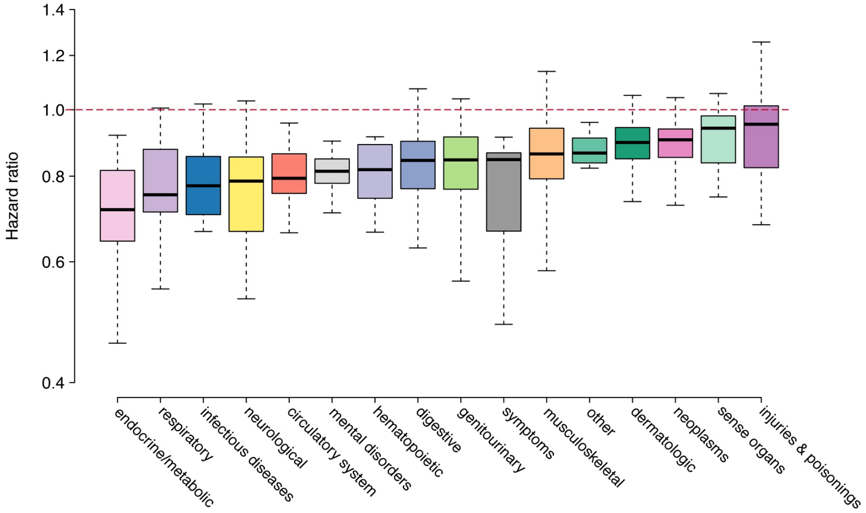

Depicted are the results of phenome-wide disease association testing with accelerometer measured moderate-to-vigorous physical activity (MVPA) as the exposure of interest, in multivariable adjusted Cox proportional hazards models not including body mass index, systolic blood pressure, diastolic blood pressure, and anti-hypertensive use as covariates. Outcomes were defined using hospital data only (i.e., not considering general practitioner data). **Panel a** plots the negative log10 p-value for the association between measured MVPA and each individual disease (grouped by category on the x-axis), with darker shaded points meeting significance at a false discovery rate of 1% (threshold depicted by horizontal dashed red line). Upward facing triangles represent higher risk (hazard ratios > 1), while downward facing triangles represent lower risk (hazard ratio < 1). **Panel b** shows the distribution of hazard ratios observed per 150-minute increase in weekly MVPA across each disease category (x-axis), with the center line depicting the within-category median hazard ratio, the bounds of the box representing quartile 1 to quartile 3, and the whiskers extending 1.5 interquartile ranges beyond the box. Categories are arranged by increasing median hazard ratio, from lowest (left) to highest (right).

Supplementary Figure 19. Associations between measured MVPA and incident disease excluding events occurring within two years

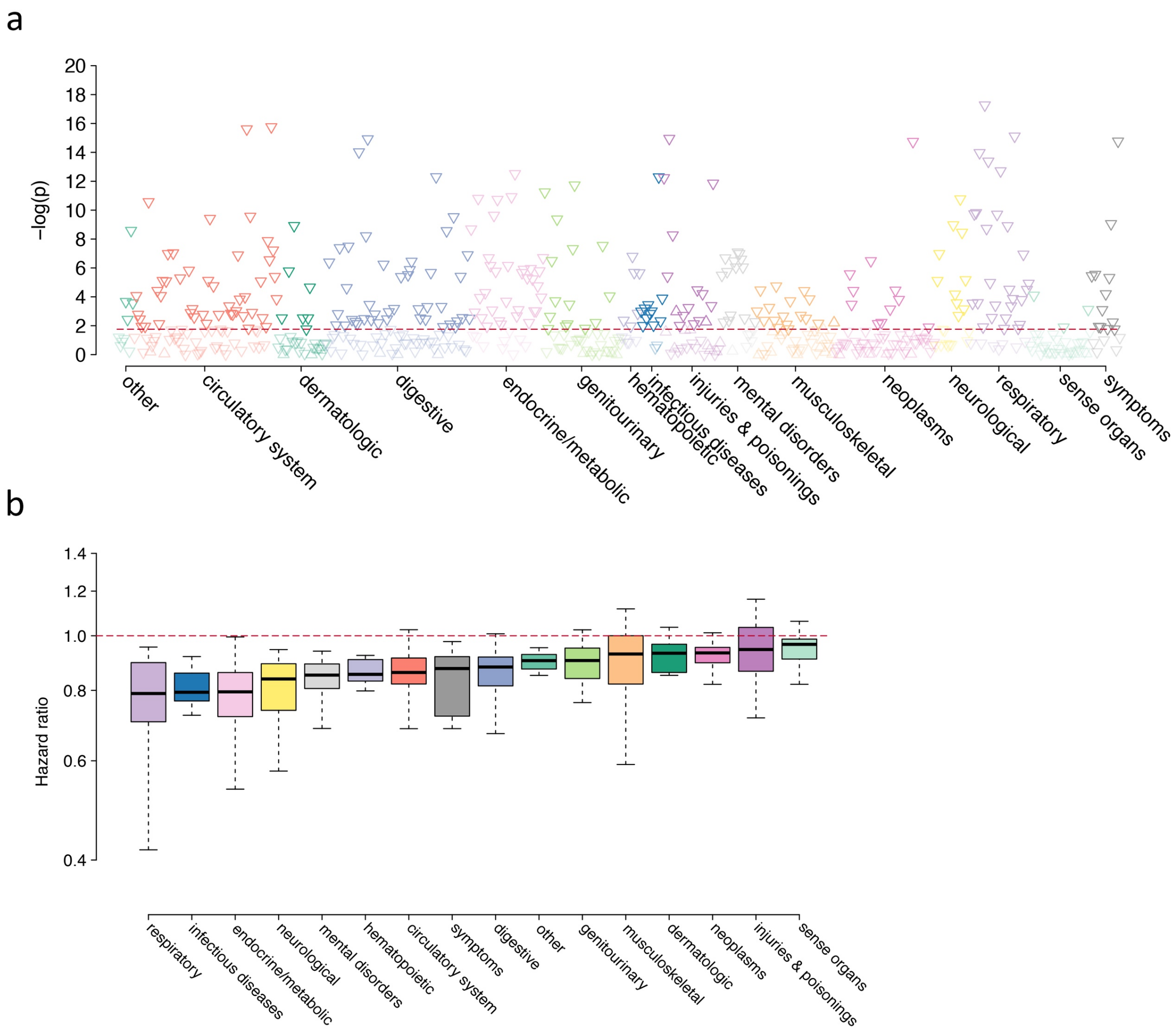

Depicted are the results of phenome-wide disease association testing with accelerometer measured moderate-to-vigorous physical activity (MVPA) as the exposure of interest, in multivariable adjusted Cox proportional hazards models. **Panel a** plots the negative log10 p-value for the association between measured MVPA and each individual disease (grouped by category on the x-axis), with darker shaded points meeting significance at a false discovery rate of 1% (threshold depicted by horizontal dashed red line). Upward facing triangles represent higher risk (hazard ratios > 1), while downward facing triangles represent lower risk (hazard ratio < 1). **Panel b** shows the distribution of hazard ratios observed per 150-minute increase in weekly MVPA across each disease category (x-axis), with the center line depicting the within-category median hazard ratio, the bounds of the box representing quartile 1 to quartile 3, and the whiskers extending 1.5 interquartile ranges beyond the box. Categories are arranged by increasing median hazard ratio, from lowest (left) to highest (right). In this landmark analysis, person-time began two years following accelerometer wear (see text).

Supplementary Figure 20. Directed acyclic graphs for primary and secondary models

a

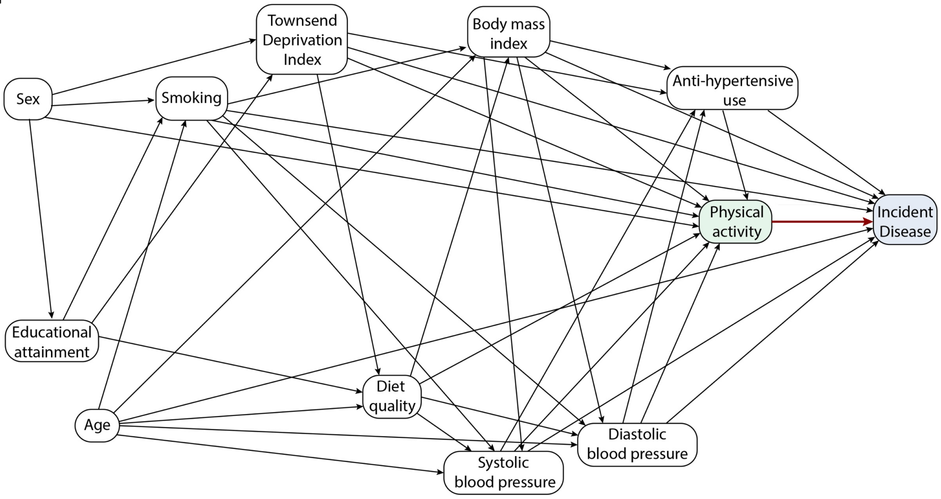

b

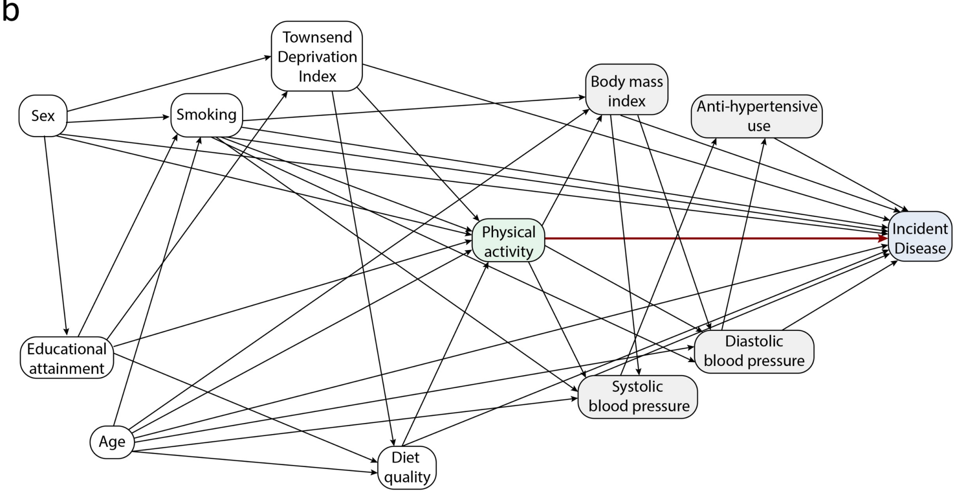

Depicted are directed acyclic graphs (DAGs) for the primary (**panel a**) and secondary (**panel b**) models assessing associations between physical activity (exposure, green) and incident disease (outcome, blue). Given our aim to broadly identify plausible associations between MVPA and disease, and our intent to assess hundreds of disease outcomes simultaneously, we selected a uniform set of potential confounding variables to adjust for in our models. Each clinical factor included in the model is depicted as a named node. In **panel b**, body mass index, systolic blood pressure, diastolic blood pressure, and anti-hypertensive use are mediators of the relationship between physical activity and incident disease, and are depicted in gray.

Supplementary References

1. Arnett, D. K. *et al.* 2019 ACC/AHA Guideline on the Primary Prevention of Cardiovascular Disease: A Report of the American College of Cardiology/American Heart Association Task Force on Clinical Practice Guidelines. *Circulation* **140**, e596–e646 (2019).
2. Piepoli, M. F. *et al.* 2016 European Guidelines on cardiovascular disease prevention in clinical practice: The Sixth Joint Task Force of the European Society of Cardiology and Other Societies on Cardiovascular Disease Prevention in Clinical Practice (constituted by representatives of 10 societies and by invited experts)Developed with the special contribution of the European Association for Cardiovascular Prevention & Rehabilitation (EACPR). *Eur Heart J* **37**, 2315–2381 (2016).
3. *Global recommendations on physical activity for health*. (WHO, 2010).
4. Hildebrand, M., VAN Hees, V. T., Hansen, B. H. & Ekelund, U. Age group comparability of raw accelerometer output from wrist- and hip-worn monitors. *Med Sci Sports Exerc* **46**, 1816–1824 (2014).
5. Khurshid, S. *et al.* Accelerometer-derived physical activity and risk of atrial fibrillation. *Eur Heart J* **42**, 2472–2483 (2021).
