# Supplementary material for: Wearable accelerometer-derived physical activity and incident disease
Source: NPJ Digit Med. 2022 Sep 2;5:131. doi: 10.1038/s41746-022-00676-9 (PMC9440134; doi:10.1038/s41746-022-00676-9)
Supplement: Supplementary file 3 — Reporting Summary [file 41746_2022_676_MOESM3_ESM.pdf]

nature portfolio

Corresponding author(s): Steven A. Lubitz

Last updated by author(s): 8/8/2022

Reporting Summary

Nature Portfolio wishes to improve the reproducibility of the work that we publish. This form provides structure for consistency and transparency in reporting. For further information on Nature Portfolio policies, see our Editorial Policies and the Editorial Policy Checklist.

Statistics

For all statistical analyses, confirm that the following items are present in the figure legend, table legend, main text, or Methods section.

n/a	Confirmed	
☐	☒	The exact sample size (n) for each experimental group/condition, given as a discrete number and unit of measurement
☐	☒	A statement on whether measurements were taken from distinct samples or whether the same sample was measured repeatedly
☐	☒	The statistical test(s) used AND whether they are one- or two-sided<br>*Only common tests should be described solely by name; describe more complex techniques in the Methods section.*
☐	☒	A description of all covariates tested
☐	☒	A description of any assumptions or corrections, such as tests of normality and adjustment for multiple comparisons
☐	☒	A full description of the statistical parameters including central tendency (e.g. means) or other basic estimates (e.g. regression coefficient) AND variation (e.g. standard deviation) or associated estimates of uncertainty (e.g. confidence intervals)
☐	☒	For null hypothesis testing, the test statistic (e.g. F, t, r) with confidence intervals, effect sizes, degrees of freedom and P value noted<br>*Give P values as exact values whenever suitable.*
☒	☐	For Bayesian analysis, information on the choice of priors and Markov chain Monte Carlo settings
☒	☐	For hierarchical and complex designs, identification of the appropriate level for tests and full reporting of outcomes
☐	☒	Estimates of effect sizes (e.g. Cohen's d, Pearson's r), indicating how they were calculated

Our web collection on statistics for biologists contains articles on many of the points above.

Software and code

Policy information about availability of computer code

Data collection	UK Biobank, a prospective cohort study whose data are publicly available for research use by application (https://www.ukbiobank.ac.uk/enable-your-research/register).
Data analysis	Data processing scripts used to perform the analyses described herein can be found at (https://github.com/shaankhurshid/acceleration_phewas). Detailed summary data supporting major study results are provided in the Supplementary Data.

For manuscripts utilizing custom algorithms or software that are central to the research but not yet described in published literature, software must be made available to editors and reviewers. We strongly encourage code deposition in a community repository (e.g. GitHub). See the Nature Portfolio guidelines for submitting code & software for further information.

Data

Policy information about availability of data

All manuscripts must include a data availability statement. This statement should provide the following information, where applicable:

- Accession codes, unique identifiers, or web links for publicly available datasets
- A description of any restrictions on data availability
- For clinical datasets or third party data, please ensure that the statement adheres to our policy

UK Biobank data are freely available for research purposes by application (https://www.ukbiobank.ac.uk/enable-your-research/register).

Field-specific reporting

Please select the one below that is the best fit for your research. If you are not sure, read the appropriate sections before making your selection.

☒ Life sciences ☐ Behavioural & social sciences ☐ Ecological, evolutionary & environmental sciences

For a reference copy of the document with all sections, see nature.com/documents/nr-reporting-summary-flat.pdf

Life sciences study design

All studies must disclose on these points even when the disclosure is negative.

Sample size	Measured activity sample (n=96,244)<br>Self-reported activity sample (n=456,374)
Data exclusions	See Figure 1<br>Measured activity: insufficient wear time, failure of calibration, withdrawn consent, missing covariates<br>Self-reported activity: withdrawn consent, missing covariates
Replication	Measured activity associations compared against self-reported activity associations
Randomization	Retrospective analysis of prospectively collected data
Blinding	N/A

Reporting for specific materials, systems and methods

We require information from authors about some types of materials, experimental systems and methods used in many studies. Here, indicate whether each material, system or method listed is relevant to your study. If you are not sure if a list item applies to your research, read the appropriate section before selecting a response.

Materials & experimental systems

n/a	Involved in the study	
☒	☐	Antibodies
☒	☐	Eukaryotic cell lines
☒	☐	Palaeontology and archaeology
☒	☐	Animals and other organisms
☐	☒	Human research participants
☐	☒	Clinical data
☒	☐	Dual use research of concern

Methods

n/a	Involved in the study	
☒	☐	ChIP-seq
☒	☐	Flow cytometry
☒	☐	MRI-based neuroimaging

Human research participants

Policy information about studies involving human research participants

Population characteristics	See Table 1.<br>Measured activity sample: Mean age 62, 56% female, 97% white<br>Self-reported activity sample: Mean age 57, 54% female, 95% white
Recruitment	9.2 million individuals aged 40-69 years living within 25 miles of 22 assessment centers in the UK were invited, and 5.4% participated in the baseline assessment. Questionnaires and physical measures were collected at recruitment, and all participants are followed for outcomes through linkage to national health-related datasets.
Ethics oversight	All participants provided written informed consent. The UK Biobank was approved by the UK Biobank Research Ethics Committee (reference# 11/NW/0382). Use of UK Biobank data (application 17488) was approved by the local Mass General Brigham Health Institutional Review Board.

Note that full information on the approval of the study protocol must also be provided in the manuscript.

Clinical data

Policy information about clinical studies

All manuscripts should comply with the ICMJE guidelines for publication of clinical research and a completed CONSORT checklist must be included with all submissions.

Clinical trial registration	N/A
Study protocol	N/A
Data collection	Questionnaires and physical measures were collected at recruitment, and all participants are followed for outcomes through linkage to national health-related datasets.
Outcomes	We defined diseases using v1.2 of the Phecode Map,40 a set of 1,867 disease definitions arranged into clinically meaningful groups and identified using standardized sets of International Classification of Disease, 9th and 10th revision codes. Diagnostic code sources included hospital data through linkage to national health-related datasets, as well as outpatient general practitioner visit data through linkage to electronic health records. The Phecode map can be found at https://phewascatalog.org/phecodes_icd10.
